# Impacts of Short-Term Antibiotic Withdrawal and Long-Term Judicious Antibiotic Use on Resistance Gene Abundance and Cecal Microbiota Composition on Commercial Broiler Chicken Farms in Québec

**DOI:** 10.3389/fvets.2020.547181

**Published:** 2020-12-21

**Authors:** Catherine Turcotte, Alexandre Thibodeau, Sylvain Quessy, Edward Topp, Guy Beauchamp, Philippe Fravalo, Marie Archambault, Marie-Lou Gaucher

**Affiliations:** ^1^Research Chair in Meat Safety, Département de Pathologie et Microbiologie, Faculté de Médecine Vétérinaire, Université de Montréal, Saint-Hyacinthe, QC, Canada; ^2^London Research and Development Centre, Agriculture and Agri-Food Canada, London, ON, Canada; ^3^Department of Biology, University of Western Ontario, London, ON, Canada; ^4^Faculté de Médecine Vétérinaire, Université de Montréal, Saint-Hyacinthe, QC, Canada; ^5^Pôle Agroalimentaire du Cnam, Conservatoire National des Arts et Métiers, Paris, France; ^6^Swine and Poultry Infectious Diseases Research Centre, Département de Pathologie et Microbiologie, Faculté de Médecine Vétérinaire, Université de Montréal, Saint-Hyacinthe, QC, Canada

**Keywords:** resistance gene, microbiota, judicious antibiotic use, health, commercial broiler chickens, conventional program, drug-free program, antibiotic withdrawal

## Abstract

The ever-increasing problem of antibiotic resistance makes routine use of antibiotics in animal production no longer considered as a reasonable and viable practice. The Chicken Farmers of Canada have developed and are implementing an Antimicrobial Use Reduction Strategy, which has the ultimate goal of eliminating the preventive use of medically important antibiotics in broiler chicken and turkey production. However, very little is known about the real overall impact of an antibiotic use reduction strategy in complex ecosystems, such as the bird intestine or the commercial broiler chicken farm. The main objectives of the present study were to compare the abundance of antibiotic resistance-encoding genes, characterize the intestinal microbiota composition, and evaluate the presence of *Clostridium perfringens*, in six commercial poultry farms adopting short-term antibiotic withdrawal and long-term judicious use strategy. Implementing an antibiotic-free program over a 15-months period did not reduce the abundance of many antibiotic resistance-encoding genes, whereas the judicious use of antibiotics over 6 years was found effective. The short-term antibiotic withdrawal and the long-term judicious use strategy altered the intestinal microbiota composition, with the *Ruminococcaceae* and *Lachnospiraceae* families being negatively impacted. These findings are in agreement with the lower production performance and with the increased *C. perfringens* populations observed for farms phasing out the use of antibiotics. Adopting a conventional rearing program on commercial broiler chicken farms selected for specific antibiotic resistance-encoding genes in many barns. This study highlights the potential impacts of different rearing programs in poultry production and will help guide future policies in order to reduce the use of antibiotics while maintaining production performance.

## Introduction

In animal husbandry, antibiotics are used to prevent and to treat infections, as growth promoting claims for antibiotics are no longer permitted in Canada (1). However, the ever-increasing problem of antibiotic resistance makes the routine use of these medicines in animal production no longer considered as a responsible approach (2). To mitigate the development of antibiotic resistance, the commitment of stakeholders coming from different sectors, such as governmental agencies, the food-producing animal industry, and the medical field involving veterinarians and physicians is essential (3). In order to guide the veterinary use of antibiotics and to preserve the effectiveness of these compounds, the World Health Organization established the List of Critically Important Antimicrobials for Human Medicine in 2005 and is reviewed periodically (4). Comprising three different categories, this list classifies antibiotics as being important, highly important, or critically important for human health (4). Canada has established criteria for the categorization of antibiotics, from category 1 to category 4, according to their decreasing human medical importance (5). In September 2017, Canada launched its pan-Canadian action plan, which aims to harmonize the actions of all stakeholders who have a role to play in addressing the antimicrobial resistance problem (6). The Chicken Farmers of Canada are implementing an Antimicrobial Use Reduction Strategy designed to eliminate the preventive use of medically important antibiotics in broiler chicken and turkey productions (7). The preventive use of category 1 antibiotics was voluntarily banned in May 2014, followed by a prohibition on the use of category 2 antibiotics since the end of 2018. Furthermore, the ban on the preventive use of category 3 antibiotics is to enter into force for Canadian poultry producers at the end of 2020, but this date is currently being reviewed. When monitoring the impacts of these voluntary changes in antimicrobial use at the farm, slaughterhouse, and retail levels, available data from the Canadian Integrated Program for Antimicrobial Resistance Surveillance is extremely useful. Surveillance data revealed that stopping the preventative use of ceftiofur, a third-generation cephalosporin, in Canadian hatcheries was associated with a lower prevalence of *Escherichia coli* and *Salmonella* isolates resistant to ceftriaxone, an antimicrobial compound belonging to the same class of antibiotics (8). In addition, previously in Europe, several countries banned the use of non-essential antibiotics in animal production, such as growth promoters in order to reduce the selection of resistance genes forming the farm resistome. In Denmark, withdrawal of antibiotics as growth promoters has been associated with a decrease in antibiotic resistance in *Enterococcus feacium* chicken broiler isolates (9). Although these are encouraging observations in targeted indicator bacteria, the global impact of an antibiotic use reduction strategy in complex ecosystems, such as the bird intestine, or the commercial broiler chicken farm remains to be better documented.

The implementation of the Chicken Farmers of Canada's Antimicrobial Use Strategy has been associated with various challenges including production losses and disease issues, such as necrotic enteritis caused by *Clostridium perfringens*. Thus, the identification of antibiotic alternative strategies to keep disease challenges under control and to maintain production performances is essential (10). To date, none of the available alternatives has proven to be as effective as antibiotics in maintaining avian gut health on commercial farms and their contribution to the fight against antibiotic resistance is still to be documented. The contribution of antibiotics to the long-term shaping of microbial communities and to the resistome of the intestine of commercial birds and consequently of poultry houses needs to be better described. Understanding of antibiotic involvement would allow a proper assessment of the global impacts of the Chicken Farmers of Canada's Antimicrobial Use Reduction Strategy and to identify valuable replacement options.

A previous study conducted by our group on different commercial broiler chicken farms aimed to compare a conventional rearing program including an antibiotic and anticoccidial-based diet to a drug-free program that was implemented over a 15-months period. In the absence of in-feed antibiotics and anticoccidials, different alternatives were used including essential oil-based products added to the feed, organic and inorganic acids in the drinking water, and a coccidiosis vaccination approach at the hatchery level (10). Rearing broiler chickens using this drug-free program significantly impacted production performance, the frequency of occurrence of necrotic enteritis, and the abundance and richness of the *C. perfringens* populations (2, 10). Now, 6 years after the close of this field study, some of the participating farms are using antibiotics judiciously, whereas some other farms went back to a conventional rearing program after completion of the 15-months study period. Thus, there is now the opportunity to revisit these farms and compare the impacts of varied antibiotic use settings in a commercial context.

The objectives of the present study were to evaluate the abundance of antibiotic resistance-encoding genes, the presence of *C. perfringens*, and the composition of the intestinal microbiota in commercial poultry farms adopting either short-term antibiotic withdrawal (15 months) or long-term judicious antibiotic use strategy (6 years).

## Materials and Methods

### Study Design

The farm selection was based on a previous study conducted by our group (10). Six (defined herein as farms A, B, C, D, E, and F) of the eight farms that took part of a previous 15-months study conducted 6 years ago agreed to participate in the current study.

In July 2012, at the end of the 15-months study, four farms (designated as farms C, D, E, and F) decided to reintroduce a conventional program (using antibiotics) in their drug-free barn (designated as “reintroduced” throughout the text), while the control barn on those farms was kept on a conventional program during both the 15-months study period and thereafter (designated as “continued” throughout the text). Those farms were then considered as having undertaken a short-term antibiotic withdrawal. The two other farms (designated as farms A and B or as “judicious” throughout the text) moved on from a conventional rearing program and from a drug-free rearing program in their control and test barns, respectively, to a program for responsibly using antibiotics in both rearing facilities, meaning that antibiotics were kept only as a therapeutic option for birds when needed (Supplementary Figure 1).

### Sample Collection

At the end of the 15-months study conducted between May 2011 and July 2012 (designated as sampling time point one throughout the text), 12 birds were randomly selected from each of the 12 participating barns, for a total of 144 birds. Birds harvested at the end of the rearing cycle were euthanized by cervical dislocation. The cecal content of the birds was sampled directly on the farm, frozen in liquid nitrogen, and transported to the laboratory. Samples were stored at −80°C for further analysis.

The same 12 barns were visited a second time in autumn 2018 (designated as sampling time point two throughout the text) for cecal sampling at the end of the rearing period. Using the same sampling protocol, 12 birds were randomly selected from each barn, for a total of 144 birds.

This protocol was approved by the Comité d'Éthique sur l'Utilisation des Animaux (CÉUA) of the Faculté de Médecine Vétérinaire of the Université de Montréal (project number 19-Rech-1970).

### DNA Extraction From Cecal Samples

In a 2-ml screw cap tube containing 500 mg of 0.1-mm silica spheres (MP Biomedical, Solon, OH, USA), 200 mg of cecal content, and 700 μl of lysis buffer [Tris-HCl 500 mM pH 8, EDTA 100 mM pH 8, NaCl 100 mM, SDS 1% (w/v)] were mixed together. A 900-μl volume of lysis buffer was used as a negative control. A mechanical lysis step was performed using a FastPrep-24™ 5G Instrument (MP Biomedical) for three runs of 60 s each, at 6 m/s. Samples were kept on ice during 5 min between each run. A second step involving thermal lysis was carried out on the samples that were heated for 20 min at 95°C and kept for 5 min on ice at the end of the procedure. The supernatant was collected after a centrifugation at 18,000 × *g* for 15 min and a standard phenol/chloroform purification protocol was used to complete the DNA extraction (11). The DNA concentration of each sample was measured using a QFX Fluorometer (Froggabio, Toronto, ON), and the purity of those samples was assessed using a Nanodrop 1000 (Fisher, Ottawa, ON) device. DNA samples were stored at −20°C until analysis.

### Detection of Gene Targets

DNA samples were screened for the presence of 12 antibiotic resistance genes for which the selection was either based on the use of antibiotics in commercial broiler chicken flocks in Canada or according to their importance for human medicine. The presence of the genes encoding the *C. perfringens* alpha toxin (*plc*) and enterotoxin (*cpe*) was also investigated in order to evaluate the impact of a short-term antibiotic withdrawal and of a long-term judicious use strategy on the presence of this animal and zoonotic pathogen. A total of 14 genes were investigated using different protocols (Supplementary Table 1). All gene targets were PCR amplified in a 25-μl reaction with 2.5 μl of 10 × PCR buffer (Biobasic, Markham, ON), 0.2 μM of dNTPs (Biobasic), 1.5 or 2 mM of MgSO_4_ (Biobasic), 1 or 1.25 U of Taq DNA Polymerase High Purity (Biobasic), template DNA, and different concentrations of specific primers (Invitrogen/Life Technologies, Burlington, ON). A Mastercycler® nexus thermocycler (Eppendorf Canada, Mississauga, ON), was used to carry out amplification reactions using cycling conditions as presented in Supplementary Table 1. A volume of 10 μl of each PCR product was subjected to gel electrophoresis using a 0.7–2% agarose gel (agarose concentration was established according to gene size) containing 0.01% SYBR Safe DNA gel strain (Fisher, Ottawa, ON). The PCR product was visualized under UV light using a 100-bp DNA ladder (Track it; Fisher).

Bacterial strains used as positive controls were grown overnight on 5% sheep blood agar plates (Fisher, Ottawa, ON) at 37°C under aerobic conditions for *Enterococcus faecium* [positive for *erm*(B) encoding for a 23S rRNA méthylase (12) and *vat*(D) encoding for a streptogramin acetyltransferase (13)], *E. faecium* [positive for *erm*(B) and *vat*(E) encoding for a streptogramin acetyltransferase (13)], *Salmonella* Heidelberg [positive for *Intl1* encoding for a class 1 integron-integrase (14)], *Enterococcus faecalis #7* [positive for *lnu*(B) encoding for a lincosamide nucleotidyltransferase (15)], and *Escherichia coli* ECL21264 [positive for *sul1* encoding for a dihydropteroate synthase (16)]. Under anaerobic conditions (AnaeroGen sachet, Fisher), *C. perfringens* c1261_A [positive for *bcrABDR* genes encoding for an ABC transporter and an overproduced undecaprenol kinase (17)] and *C. perfringens* AHL 155 (positive for *plc* and *cpe* genes) were grown overnight on 5% sheep blood agar plates (Fisher, Ottawa, ON) at 37°C. For DNA extraction, five colonies were suspended in 50 μl of a 6% Chelex solution (Bio Rad, Saint-Laurent, QC), heated at 56°C for 25 min and at 95°C for 10 min. The DNA-containing supernatant was collected after centrifugation at 18,000 × *g* for 5 min and used in PCR reactions. The positive control used for the *mcr-1* gene [encoding for a phosphoethanolamine transferase (18)] PCR amplification was DNA extracted from a French livestock *E. coli* strain expressing both a phenotype and a genotype of colistin resistance (19). The positive controls used for the PCR detection of *vga*(A) [encoding for ATP-binding proteins in active efflux (12)] and *vgb*(A) [encoding for a hydrolase (12)] was the plasmid pBluescript II SK+ (Biobasic) including the DNA fragment amplified with the primers of the resistance gene target.

### Quantification of Resistance Gene Targets

The abundance of selected resistance gene targets was determined by qPCR as previously described (20–22). Gene targets *bcrA, bcrB, lnu*(B), and *vat*(E) genes were quantified using a Roche LC96 Real Time PCR thermocycler (Roche Canada, Laval, QC) with LightCycler® 96 System Software, version 1.1. The gene targets *erm*(B), *intl1*, and *sul1* were quantified using a Bio-Rad CFX96 real-time PCR instrument with Bio-Rad CFX Manager software, version 3.1. Primers (Invitrogen/Life Technologies), hydrolysis probes (Sigma-Aldrich, Toronto, ON), and cycling conditions are listed in Supplementary Table 2. Reactions were performed in 25-μl reaction volumes using the Brilliant II QPCR Master Mix (Agilent, Toronto, ON) for the TaqMan PCR and the Brilliant II SYBR Green® Low ROX QPCR Master Mix (Agilent) for the SYBR Green PCR (Agilent). Two microliters of DNA template (10 ng of DNA) was added to each reaction, and sterile water was used to reach the final volume. Each reaction, including the negative control, was run in triplicate.

The abundance of each gene in all experimental samples was determined using a standard curve. For the *erm*(B), *intl1*, and *sul1* gene targets, respectively, the DNA fragment amplified with the primers of the gene target was cloned into the pSC-A-amp/kan plasmid using the StrataClone PCR Cloning kit (Agilent) and following the manufacturer's instructions before being used to transform *E. coli* competent cells from the StrataClone SoloPack (Agilent).

For *bcrA, bcrB, lnu*(B), and *vat*(E) gene targets, each standard curve was constructed using the plasmid pBluescript II SK+ (Biobasic) including the DNA fragment amplified with the primers of the gene target. For purification, the plasmid was linearized with the Not1-HF enzyme (New England Biolabs, Whitby, ON) for 2 h at 37°C and ran on a 1.5% agarose gel with SYBR Safe DNA gel stain (Fisher). The linearized plasmid was recovered using the QIAquick gel extraction kit (Quiagen, Montréal, QC). The plasmid DNA concentration was measured using a QFX Fluorometer (Froggabio), and the number of plasmid copies was calculated. The plasmid was diluted using a 10-fold serial dilution approach, and these dilutions were used for the standard curve construction.

### 16S rRNA Gene Amplicon Sequencing

Sequencing of the V4 region of the 16S rRNA gene was performed using the Illumina MiSeq platform. DNA was extracted from the cecal contents of all 288 birds. The 144 samples from the sampling time point one were multiplexed with controls for sequencing in one lane. The 144 samples from the sampling time point two and controls were sequenced in a separate lane. Libraries were prepared using a Mastercycler® nexus (Eppendorf Canada) with the forward primer 5′-ACACTGACGACATGGTTCTACAGTGCCAGCMGCCGCGGTAA-3′ and the reverse primer 5′-TACGGTAGCAGAGACTTGGTCTGGACTACHVGGGTWTCTAAT-3′ (Invitrogen/Life Technologies) (23). Following the manufacturer's instructions with some modifications, the amplification of the 292-bp segment was performed using 6 μl of 5 × SuperFi™ Buffer (Fisher, Ottawa, Ontario), 6 μl of 5 × SuperFi™ GC Enhancer (Fisher), 0.6 μl of 10 mM dNTP mix (Fisher), 0.9 μl of 20 μM primers (Invitrogen/Life Technologies), 0.6 μl of 20 mg/ml Pierce™ bovine serum albumin (Fisher), 0.3 μl of 2 U/μl Platinum SuperFi DNA Polymerase (Fisher), and 1.5 μl of DNA (15 ng) for a total reaction volume of 30 μl. Total volume was completed with sterile water. Sterile water was used as negative control, and the ZymoBIOMICS Microbial Community DNA Standard (Cedarlane, Burlington, ON) was used as positive control. Cycling conditions were as follows: a hot start step of 5 min at 95°C, followed with 23 cycles of 30 s at 95°C, 30 s at 55°C, and 3 min at 72°C, and a final elongation step of 10 min at 72°C. A volume of 10 μl of the PCR product was submitted to electrophoresis using 1.5% agarose gel containing 0.01% SYBR Safe DNA gel strain (Fisher). The PCR product was visualized under UV light using a 1-kb DNA ladder (Track it; Fisher).

Two libraries were prepared and sequenced separately. The first library was made up of 144 samples, six negative controls (one for each farm to validate the quality of the DNA extraction procedure), one negative control (sterile water), and one positive control (ZymoBIOMICS Microbial Community DNA Standard). The second library consisted of 144 samples, six negative controls (one for each farm to validate the quality of the DNA extraction procedure), two negative controls (sterile water), and one positive control (ZymoBIOMICS Microbial Community DNA Standard). Libraries were sent to the Génome Québec Innovation Centre (Montreal, QC) for DNA sequencing using an Illumina MiSeq PE250 platform (Illumina, San Diego, CA, USA).

As previously described by Larivière-Gauthier et al., with some modifications, the obtained sequences were cleaned using MOTHUR v. 1.14.3 (24). Reads that were too long or ambiguous were eliminated, and the Silva database v.132 was used to align unique sequences. Chimeras were discarded using the VSEARCH tool (25), and reads were clustered into operational taxonomic units, with a 3% dissimilarity (OTUs). Mothur-formatted Ribosomal database project trainset version 16 was used to classify the obtained OTUs. Further data analysis was done using RStudio (version 1.2.5033, 2019) with the following packages: phyloseq, vegan, dplyr, scales, grid, reshape2, igraph, ape, gplots, lme4, phangorn, plotly, tidyr, data.table, Maaslin2, ggplot2, stringr, and devtools.

In order to avoid the presence of OTUs found only in a single flock, sequences that were present in more than 12 samples for each sampling time point analyzed were retained for biomarker analysis. To characterize the microbial communities associated with the different rearing programs and sampling time points, MaAsLin2 (Multivariate Association with Linear Models) was used in RStudio (26).

### Data Analysis and Statistics

For qPCR values, the detection limit for quantification was set at one copy per reaction. For values below this limit, a 0.9 gene copy value per reaction was chosen to calculate the average copy number of each sample ran in triplicate. This average was converted into a number of gene copies/ng of DNA, and resulting values were expressed on both a weight basis (raw values) and a ratio referenced to the total bacterial content of the sample according to the 16S rRNA gene copy number. GraphPad Prism (v8.0.2, GraphPad Software Inc., La Jolla, CA) was used to prepare the figures.

A first analysis using a linear mixed model measured changes in the mean of the log-transformed qPCR copy number of the 16S rRNA gene, considering the sampling time point, the rearing program, and the interaction between both as fixed effects and the farm as a random variable (27). Farms A and B were not included in this analysis as they were not using antibiotics at sampling time point two.

A second statistical analysis using a linear mixed model and considering the farm as a random effect analyzed the fixed effect of the rearing programs on the mean of the log-transformed qPCR raw values and ratios at sampling time point one for each gene target. The same model was used for sampling time point two and also excluded farms A and B that did not use antibiotics.

A third analysis using a linear mixed model measured changes in the mean of the log-transformed qPCR raw values and ratios of each gene target, considering the sampling time point and program variables as fixed effects, and the farm as a random variable. Again, farms A and B were not included in this analysis. *A priori* contrasts were performed to compare mean values at each sampling time point and to compare means at sampling points one and two among programs. For these comparisons, the Benjamini–Hochberg sequential procedure was used to adjust the alpha level downward. The familywise error rate was set at 5% (28).

A fourth analysis considered each farm separately. A linear model was used to analyze changes in the mean of the log-transformed qPCR raw values and ratios for each gene target as a function of sampling points and rearing programs, followed by the use of *a priori* contrasts, as described above.

For the 16S rRNA amplicon metagenomic sequencing analyses, the alpha and the beta diversity indices were calculated using Rstudio. For alpha diversity analyses, the richness and the evenness were measured using diversity indices of OTU observed, Shannon, and inverse Simpson. To analyze the fixed effect of the rearing program on the mean of alpha diversity indices for sampling time points one and two, a linear mixed model with the farm as a random effect was used. To measure the effect of the sampling time point on the mean of alpha diversity indices, a linear mixed model was used considering the rearing program, the sampling time point, and the interaction between both as fixed effects and the farm as a random variable. Again, farms A and B were excluded from this analysis due to their different status regarding antimicrobial use. For farms A and B, a linear mixed model was also used considering the farm as a random variable and the rearing program as a fixed effect. For both analyses, *a priori* contrasts, as described above, were used. Statistical analyses were performed using SAS v.9.4 (Cary, N.C.). For the beta diversity analysis, distances between samples were displayed by non-metric multidimensional scaling (NMDS) graphs and calculated using the Jaccard and Bray–Curtis indices (24). Statistical differences between groups were calculated using the ADONIS test, with a significance level of 0.05.

## Results

### Detection of Gene Targets

DNA samples were screened individually or as pooled samples for the presence of 14 genes. Based on the positive detection of *bcrA, bcrB, erm*(B), *intl1, lnu*(B), *sul1*, and *vat*(E), those gene targets were then quantified by qPCR (Table 1).

**Table 1:** Sample treatment and PCR detection results.

**Gene**	**Sample treatment**	**Detection result (%)**
*bcrA* *bcrB*	From 288 samples pooled in groups of 6 or 4 samples	100
*bcrR* *vat*(D)	From 288 samples pooled in groups of 4 samples	100 4
*vat*(E)		72
*mcr-1*		0
*lnu*(B)	From 288 individual samples	34
*cpe*		0
*plc*		17
*erm*(B)	From 246 individual samples	100
*sul1*	From 48 individual samples	69
*intl1*		92
*vga*(A)	From 12 individual samples	Non-specific amplification
*vgb*(A)		

No quantitative approach was performed on *bcrR, vga*(A), *vgb*(A), *vat*(D), *mcr-1, cpe*, and *plc* genes. Pools were all found positive for the presence of *bcrA, bcrB*, and *bcrR* genes. Only *bcrA* and *bcrB* were submitted to the quantitative PCR approach as the presence of the *bcrR* regulator gene is not essential in conferencing a bacitracin resistance phenotype in bacteria carrying the bacitracin resistance operon (29). Attempts to evaluate the presence of *vga*(A) and *vgb*(A) genes were also made, but non-specific amplification issues have prevented the use of a quantitative approach to describe the presence of these genes. From a total of 72 DNA pools, the *vat*(E) gene was kept for the following quantitative analyses as 72% of the pooled samples were found positive for the presence of this gene, while only 4% of the samples screened were positive for *vat*(D). All the pools screened were negative for the presence of *mcr-1* (Table 1).

#### *Clostridium perfringens* Detection Results

All the 288 individual samples screened were negative for the presence of the *cpe* gene. For the detection of the *C. perfringens* alpha toxin-encoding gene (*plc*), a total of 48 samples from the 288 tested individually were identified as positive (Table 1).

### Quantification of Resistance Gene Targets

**Figure 1:**
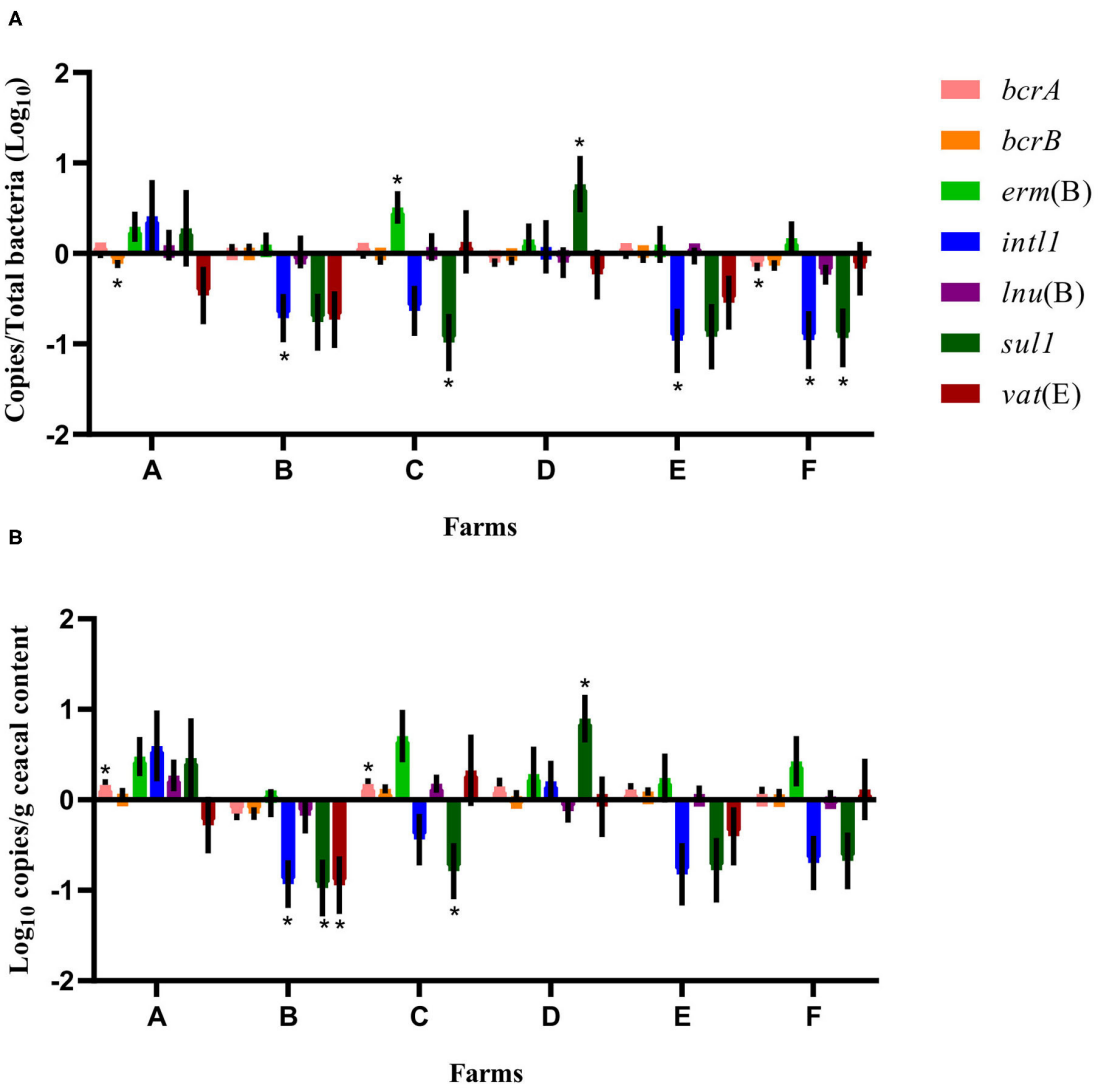
Difference of resistance gene targets between drug-free and conventional flocks for each farm at sampling time point one. Negative results indicate a decrease in gene target, and positive results indicate an increase in gene target in drug-free flocks. Data are presented as the mean (SEM) of 12 replicates. For the quantitative approach, each sample was run in triplicate (*n* = 3). **(A)** Values are expressed on a ratio referenced to the total bacterial content of samples (16S rRNA). **(B)** Values are expressed on a weight basis (raw values). *Significant values are lower than the alpha level adjusted with the Benjamini–Hochberg method.

A quantitative PCR approach was used to establish the relative abundance of *bcrA, bcrB, erm*(B), *intl1, lnu*(B), *sul1, vat*(E), and 16S rRNA gene targets. After a short-term antibiotic withdrawal of 15 months, the relative abundance of *sul1* or *intl1* or both genes, decreased significantly in the drug-free flocks of four farms out of the six sampled (Figure 1).

**Figure 2:**
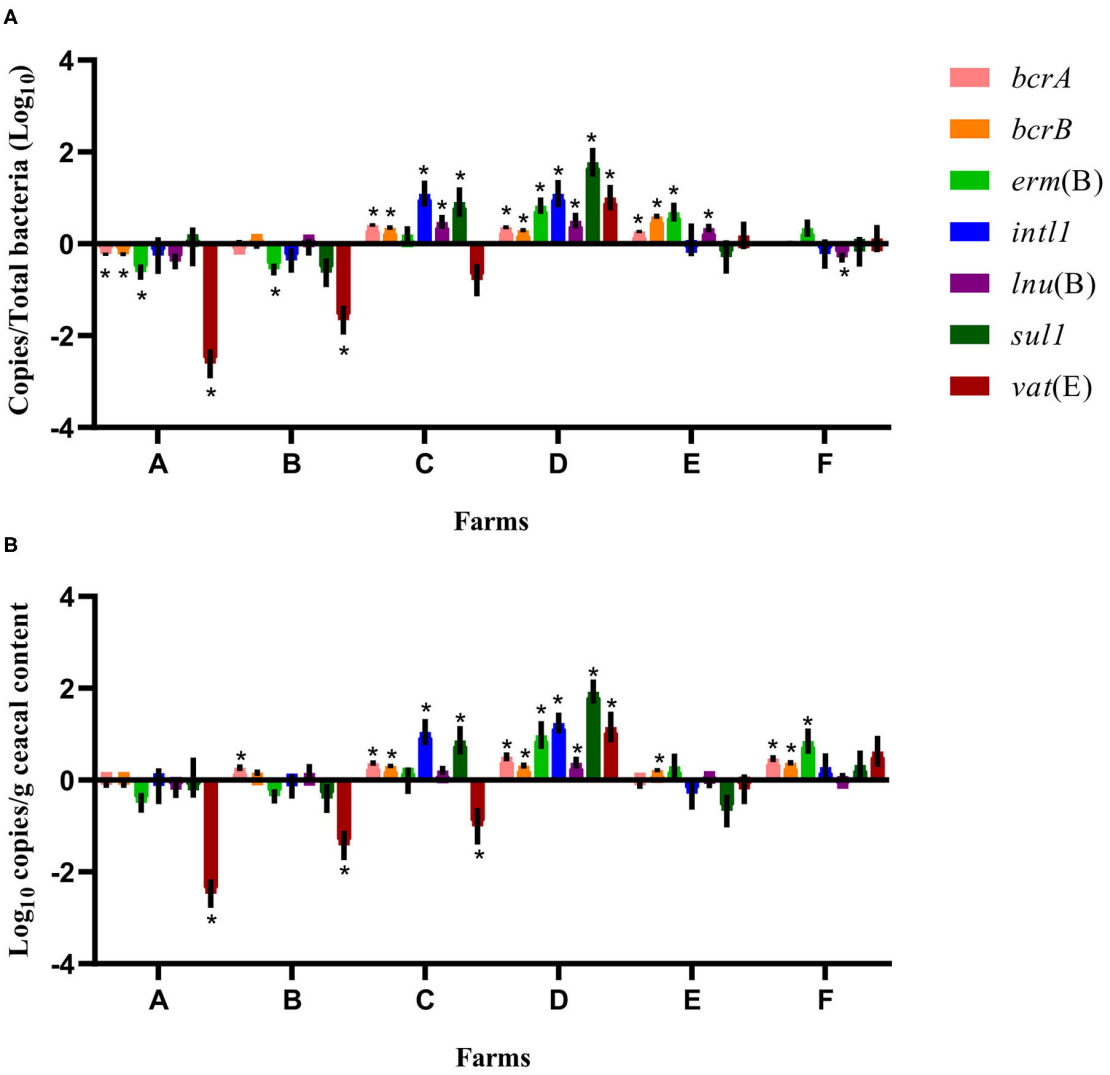
Difference of resistance gene targets between flocks sampled from conventional barns at sampling time point one and the flock from the same barn at sampling time point two. At sampling time point one, conventional barns from farms C to F remained on a conventional rearing program after the 15-months study period, whereas barns from farms A and B moved to a program for judiciously using antibiotics. Negative results indicate a decrease in gene target, and positive results indicate an increase in gene target in the sampled flock at sampling time point two. Data are presented as the mean (SEM) of 12 replicates. For the quantitative approach, each sample was run in triplicate (*n* = 3). **(A)** Values are expressed on a ratio referenced to the total bacterial content of samples (16S rRNA). **(B)** Values are expressed on a weight basis (raw values). *Significant values are lower than the alpha level adjusted with the Benjamini–Hochberg method.

**Figure 3:**
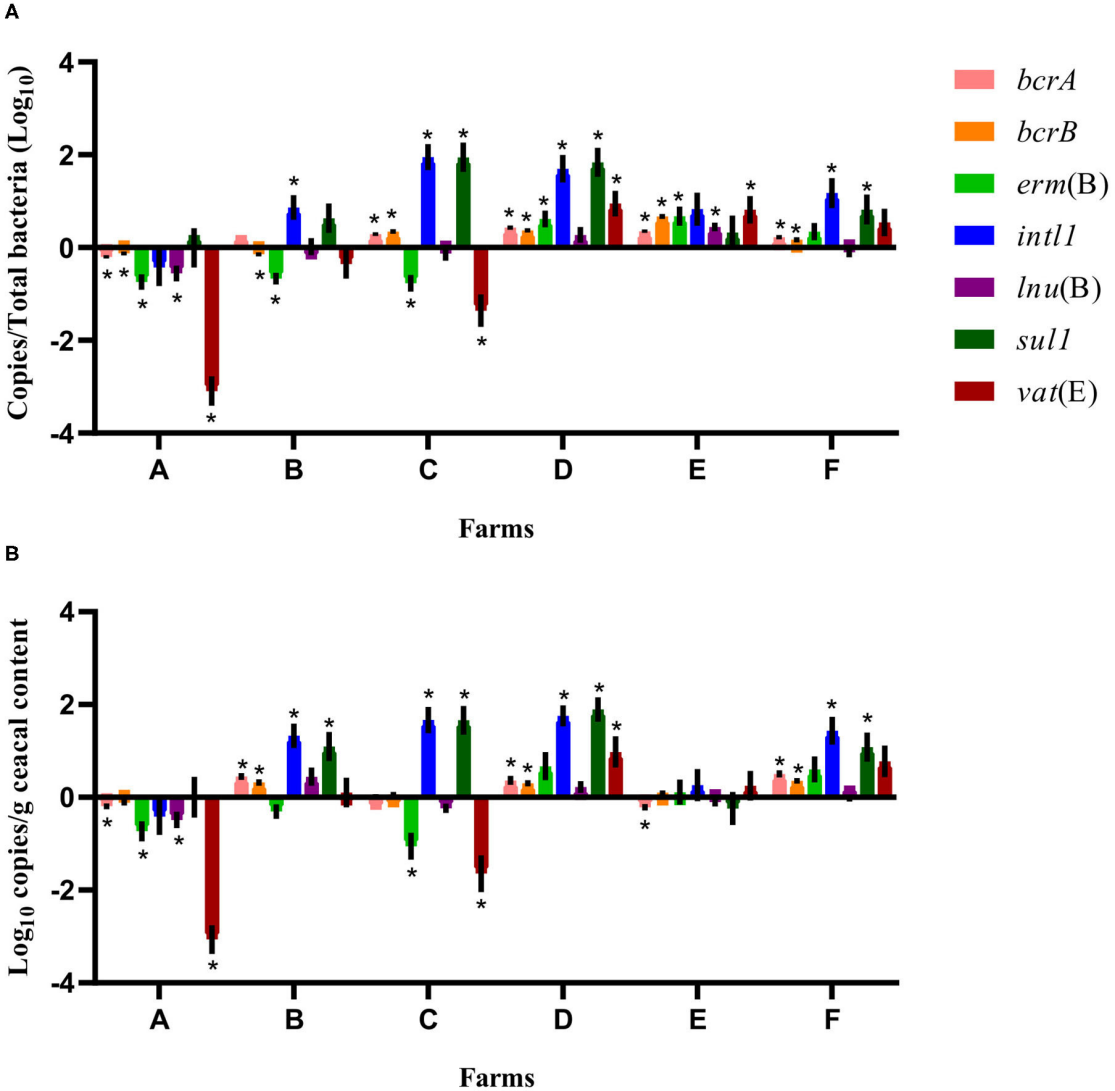
Difference of resistance gene targets between flocks sampled from drug-free barns at sampling time point one and the flock sampled from the same barn at sampling time point two. Drug-free barns from farms C to F went back to a conventional rearing protocol after the 15-months study period, whereas drug-free barns from farms A and B moved to a program for judiciously using antibiotics. Negative results indicate a decrease in gene target, and positive results indicate an increase in gene target in the sampled flock at sampling time point two. Data are presented as the mean (SEM) of 12 replicates. For the quantitative approach, each sample was run in triplicate (*n* = 3). **(A)** Values are expressed on a ratio referenced to total bacterial content of samples (16S rRNA). **(B)** Values are expressed on a weight basis (raw values). *Significant values are lower than the alpha level adjusted with the Benjamini–Hochberg method.

For some of the flocks sampled from farms A and B at sampling time point two, a long-term judicious use strategy (6 years) was associated with a decrease in the relative abundance and the absolute copy number of some antibiotic resistance-encoding genes, namely, *bcrA, bcrB, erm*(B), *lnu*(B), and *vat*(E) (Figures 2, 3). In contrast, routine use of antibiotics over a 6-years period on farms C, D, E, and F was associated, for some of the sampled flocks, with an increase in the relative abundance and in the absolute copy number of many of the resistance gene targets, namely, *bcrA, bcrB, erm*(B), *intl1, lnu*(B), *sul1*, and *vat*(E) (Figures 2, 3).

Regarding the variability of the 16S rRNA gene target abundance according to rearing programs and sampling time points, a linear mixed model showed no differences. A second analysis grouping the six conventional flocks and the six drug-free flocks at sampling time point one showed an increase in the relative abundance (*p* = 0.0074) and the absolute copy number (*p* = 0.0232) of *erm*(B) in drug-free flocks. A third analysis investigating the impacts of a short-term antibiotic withdrawal and a long-term conventional rearing program (excluding farms A and B) on the abundance of antibiotic resistance-encoding genes showed that only the abundance of *bcrB* expressed as raw values increased (*p* = 0.0082) when using a long-term conventional program.

When considering the farm as the unit of analysis, the mean abundance of each gene target was compared between the conventional and drug-free flocks at sampling time point one for each participating farm. As presented in Figure 1, the relative abundance expressed as a ratio of the antibiotic resistance gene target to the 16S rRNA content of the samples showed, for drug-free flocks, a decrease in *bcrA, intl1*, and *sul1* on farm F, of *bcrB* on farm A, of *intl1* on farms B and E, and of *sul1* on farm C. In contrast, *erm*(B) and *sul1* increased on farms C and D, respectively. In the drug-free flocks sampled, raw values revealed a decrease in *intl1, sul1*, and *vat*(E) for farm B and a decrease for *sul1* only on farm C. In contrast, *bcrA* increased for farms A and C, whereas *sul1* increased for farm D.

**Figure 4:**
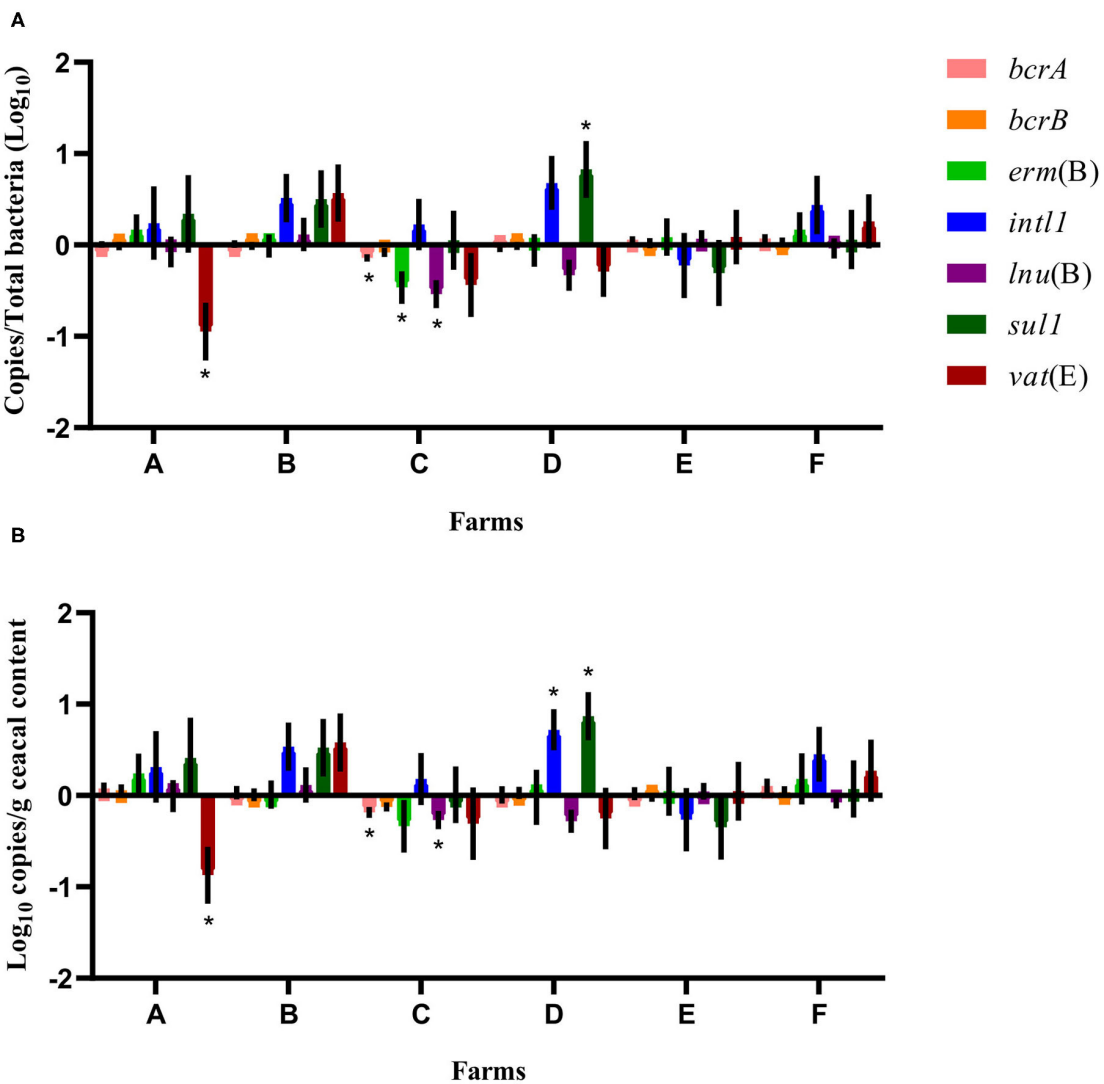
Difference of resistance gene targets at sampling time point two between flocks of the same participating farm that adopted either a conventional rearing program or a program for judiciously using antibiotics after the completion of the 15-months study period, considering the barn that was on a drug-free program during the 15-months study period as the comparison reference unit. Negative results indicate a decrease in gene target, and positive results indicate an increase in gene target in the flock sampled at sampling time point two used as a reference unit. Data are presented as the mean (SEM) of 12 replicates. For the quantitative approach, each sample was run in triplicate (*n* = 3). **(A)** Values are expressed on a ratio referenced to total bacterial content of samples (16S rRNA). **(B)** Values are expressed on a weight basis (raw values). *Significant values are lower than the alpha level adjusted with the Benjamini–Hochberg method.

Considering the farm as the unit of analysis and the barn that was on a drug-free program during the 15-months study period as the comparison reference unit, the mean relative abundance of each gene target was compared at sampling time point two between flocks of the same participating farm that had adopted either a conventional rearing program or a program for judiciously using antibiotics after the completion of the 15-months study period (Figure 4). For farms A and B, ratios and raw values obtained for the antibiotic resistance gene targets measured showed a decrease in *vat*(E) for farm A. For farms C, D, E, and F, ratios and raw values showed a decrease in *bcrA, erm*(B), and *lnu*(B) genes and for *bcrA* and *lnu*(B), respectively, on farm C. As opposed, ratios and raw values showed an increase for *sul1*, and for *sul1* and *intl1*, respectively, on farm D.

Considering the farm as the unit of analysis, the mean relative abundance of each gene target was compared between sampling time points one and two, considering two categories of barns: barns using a conventional rearing program at both sampling time points (farms C, D, E, and F) and barns moving from a conventional program at sampling time point one to a program for judiciously using antibiotics after the 15-months study period (farms A and B). In Figure 2, for farms A and B, ratios showed a decrease for *erm*(B) and *vat*(E) on both farms and of *bcrA* and *bcrB* on farm A. For farms C, D, E, and F, the relative abundance increased for *bcrA, bcrB*, and *lnu*(B) on farms C, D, and E. The *intl1* and *sul1* genes increased on farms C and D, whereas an increase in *erm*(B) and *vat*(E) was noted for farms D and E, and farm D, respectively. As opposed, *lnu*(B) decreased on farm F. For farms A and B, raw values for *vat*(E) showed a decrease on both farms, whereas raw values of *bcrA* gene increased on farm B. Raw values also showed an increase for *bcrA* on farms C, D, and F, and for *bcrB* on farms C, D, E, and F. Raw values for *intl1* and *sul1*, and for *erm*(B) all showed an increase for farms C and D, and for farms D and F, respectively. An increase in *lnu*(B) and *vat*(E) genes was only observed for farm D. In contrast, raw values showed a decrease for *vat*(E) in farm C.

Considering the farm as the unit of analysis, the mean relative abundance of each gene target was compared between sampling time points one and two, considering two categories of barns: drug-free barns at sampling time point one going back to a conventional rearing program (farms C, D, E, and F) and drug-free barns at sampling time point one moving to a program for responsibly using antibiotics (farms A and B) after the 15-months study period. As shown in Figure 3, for farms A and B, the relative abundance expressed as a ratio showed a decrease for *bcrB* and *erm*(B) on both farms and for *bcrA, lnu*(B), and *vat*(E) on farm A. In contrast, *intl1* increased on farm B. For farms C, D, E, and F, the relative abundance showed an increase for *bcrA* and *bcrB* on all four farms. Ratio values for *erm*(B) and *vat*(E) increased for farms D and E, for *intl1* and *sul1* on farms C, D, and F and for *lnu*(B) on farm E. As opposed, *erm*(B) and *vat*(E) decreased for farm C. Raw values of the antibiotic resistance gene target presented a decrease for *bcrA, erm*(B), *lnu*(B), and *vat*(E) on farm A, whereas an increase for *bcrA, bcrB, intl1*, and *sul1* was shown for farm B. For farms C, D, E, and F, raw values showed an increase for *bcrA, bcrB* on farms D and F, for *intl* and *sul1* on farms C, D, and F, and of *vat*(E) on farm D. In contrast, raw values presented a decrease for *bcrA* on farm E, and for *erm*(B) and *vat*(E) on farm C.

### 16S rRNA Gene Amplicon Metagenomic Sequencing

The two sequenced libraries, one for each sampling time point, were both analyzed at the same time on Mothur. Positive controls corresponded to the theorical composition of the ZymoBIOMICS Microbial Community DNA Standard. A total of 192, 168, and 190 sequences were obtained for the three negative controls made from sterile water. The average of the sequences obtained for the 12 negative controls from DNA extraction was 1,893 sequences and the highest and lowest numbers of sequences obtained were 11,798 and 11. Positive and negative controls were excluded for the rest of the data analysis. Among the 288 samples left, an average of 34,661 sequences were obtained per sample and a total of 37,325 OTUs were detected. The highest and lowest numbers of sequence obtained in a sample were 64,131 and 10,107. Considering the distribution of sequences, 10 samples were excluded from the analysis due to a low number of sequences (below 15,000).

Comparing the conventional and the drug-free flocks sampled at sampling time point one (Table 2), the OTU observed and the Shannon indices showed no significant difference. In contrast, according to the inverse Simpson index, conventional flocks showed a higher alpha diversity when compared to drug-free flocks.

**Table 2:** (A) Comparison by column of mean (SEM) alpha diversity indices between conventional and drug-free flocks after the 15-months study (sampling time point one) using a linear mixed model.

	**Observed**	**Shannon**	**InvSimpson**
**(A) SAMPLING TIME POINT ONE**			
Conventional	409.45 (16.99)	4.44 (0.11)	38.87 (4.95)
Drug-free	421.12 (16.99)	4.36 (0.11)	32.98 (4.95)
*p*-value	0.13	0.11	0.03
**(B) SAMPLING TIME POINT TWO**			
Judicious	494.94 (28.88)	4,19 (0.07)	24,14 (2.77)
Reintroduced	524.08 (21.11)	4,26 (0.05)	24,85 (2.20)
Continued	573.10 (21.11)	4,56 (0.05)	38.67 (2.20)
***p*****-values**			
^*^Judicious vs. reintroduced	0.42	0.40	0.84
^*^Judicious vs. continued	0.03^a^	<0.0001	<0.0001
^*^Reintroduced vs. continued	<0.0001	<0.0001	<0.0001

*(B) Comparison by column of mean (SEM) alpha diversity indices, 6 years after the 15-months study (sampling time point two), between flocks from barns that adopted a long-term judicious use strategy (judicious), flocks that continued the conventional rearing program (continued), and flocks that reintroduced antibiotics after the 15-months study (reintroduced) using a linear mixed model*.

a *Not statistically significant after the alpha level was adjusted downward*.

* *The p-values are for the indicated pairwise comparisons*.

For sampling time point two (Table 2), the alpha diversity indices were compared between flocks sampled from barns using antibiotics judiciously, and barns that continued and reintroduced the antibiotics after the 15-months study. All indices showed that the conventional flocks that kept using a conventional rearing protocol after the 15-months study period had a higher alpha diversity than flocks that reintroduced antibiotics after a short-term antibiotic withdrawal. In addition, considering the Shannon and inverse Simpson indices, conventional flocks still using a conventional rearing protocol after the 15-months study period had a higher alpha diversity than flocks from farms that adopted a long-term program for judiciously using antibiotics. No differences were noted between flocks that reintroduced antibiotics after a short-term antibiotic withdrawal and flocks from farms A and B that moved to a long-term program for responsibly using antibiotics.

The alpha diversity indices were compared between the two sampling time points (Table 3). For all farms, results showed that the richness at sampling time point two, according to the OTU observed index, was greater than the sample diversity observed at sampling time point one. In contrast, the inverse Simpson index showed that the alpha diversity was greater at sampling time point one than at sampling time point two for farms A and B (Table 3).

**Table 3:** (A) Comparison by column of mean (SEM) alpha diversity indices, for farms C, D, E, and F, between barns sampled after the 15-months study (sampling time point one) and 6 years later (sampling time point two) using a linear mixed model.

	**Observed**	**Shannon**	**InvSimpson**
**All sampling time points**			
**(A) FARMS C, D, E, AND F**			
**Sampling time point one**			
Conventional	419.17 (17.56)	4.49 (0.09)	40.36 (4.40)
Drug-free	415.71 (17.56)	4.36 (0.09)	31.40 (4.40)
**Sampling time point two**			
Continued	573.10 (17.46)	4.56 (0.09)	38.67 (4.38)
Reintroduced	524.08 (17.46)	4,26 (0.09)	24,85 (4.38)
***p*****-value**			
^*^Conventional vs. continued	<0.0001	0.53	0.72
^*^Drug-free vs. reintroduced	<0.0001	0.32	0.16
**(B) FARMS A AND B**			
**Sampling time point one**			
Conventional	393.05 (13.71)	4.35 (0.16)	35.89 (8.48)
Drug-free	438.05 (13.71)	4.36 (0.16)	36.92 (8.48)
**Sampling time point two**			
Judicious^a^	492.58 (13.13)	4.22 (0.16)	25.93 (8.42)
Judicious^b^	497.29 (13.13)	4.17 (0.16)	22.34 (8.42)
***p*****-value**			
^*^Conventional vs. judicious^a^	<0.0001	0.21	0.04
^*^Drug-free vs. judicious^b^	0.002	0.05	0.003

*(B) Comparison by column of mean (SEM) alpha diversity indices, for farms A and B, between barns at sampling time points one and two, using a linear mixed model*.

a *Was on a conventional rearing program at sampling time point one*.

b *Was on a drug-free program at sampling time point one*.

* *The p-values are for the indicated pairwise comparisons*.

**Figure 5:**
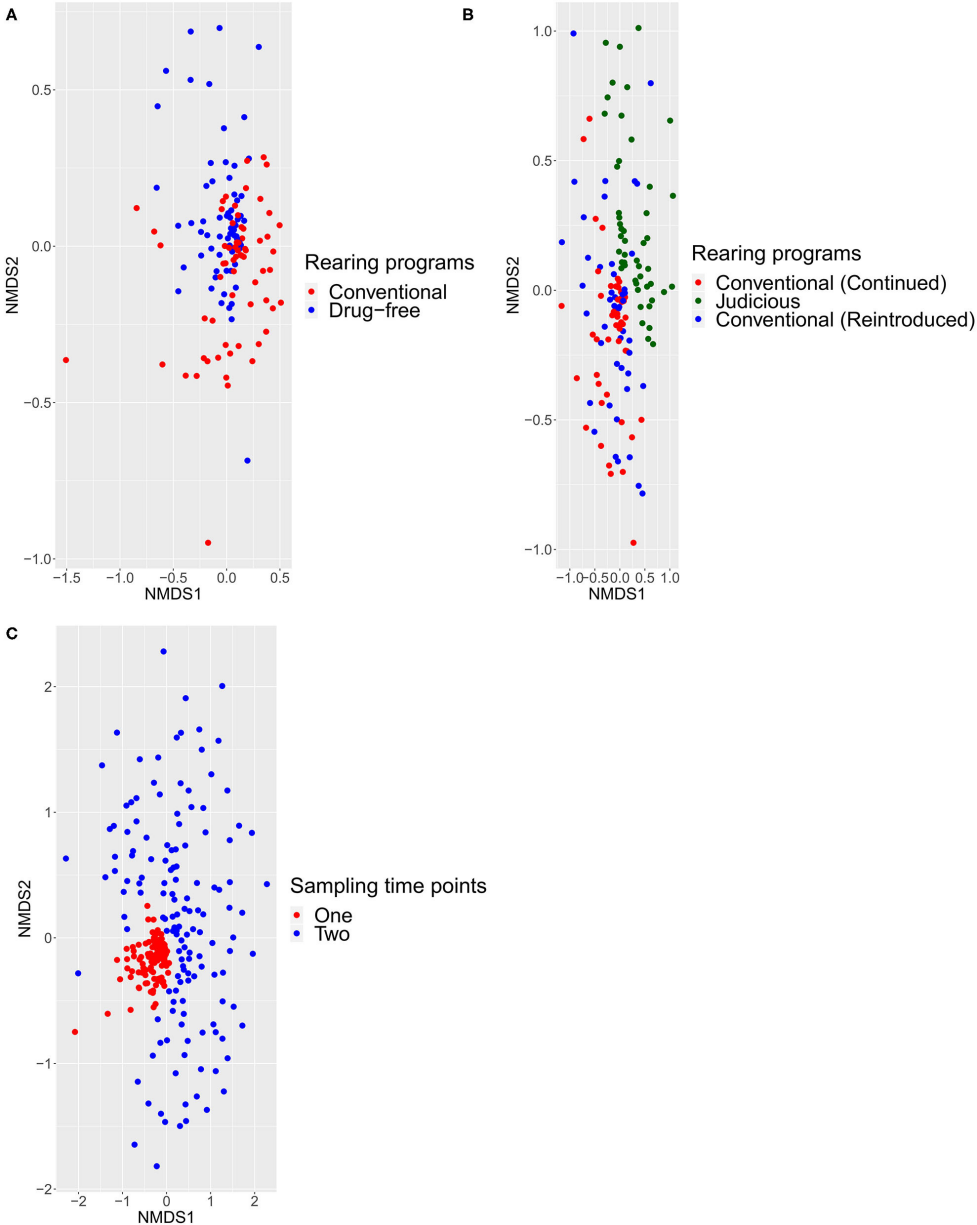
Beta diversity calculated with the Jaccard index using ADONIS test with a significance level of 0.05. All comparisons were statistically significant (*p* < 0.0001). Each point represented one bird sampled. **(A)** Differences between conventional and drug-free programs at sampling time point one. **(B)** Difference between flocks, at sampling time point two, from barns that adopted a long-term strategy for judiciously using antibiotics, barns that continued the conventional rearing program and barns that reintroduced antibiotics after a short-term antibiotic withdrawal. **(C)** Differences between sampling time points one and two.

For beta-diversity, sampled flocks were compared according to different rearing programs and sampling time points and visualized with an NMDS. Using the Jaccard (Figure 5) and Bray–Curtis (Supplementary Figure 2) indices, the ADONIS test was performed. Comparing the conventional and drug-free flocks at sampling time point one, the distance matrix showed the presence of two distinct groups. For sampling time point two, the NMDS showed a distinct structure for flocks sampled from barns that adopted a long-term strategy for judiciously using antibiotics, flocks that continued the conventional rearing program, and flocks that reintroduced antibiotics after a short-term antibiotic withdrawal. In addition, the beta-diversity was measured between all samples from the two sampling time points. Regardless of the rearing program, samples collected from sampling time point one showed a pattern of aggregation, while samples collected at sampling time point two presented a scattered profile. Finally, for each distance matrix, using a different scale, it was possible to distinguish each sampled flock from a sampled farm and each farm from one another.

In order to identify associations with biomarkers, MaAsLin2 was used according to the rearing program at sampling time point one (conventional and drug-free barns) and two (continued, reintroduced, judicious barns). For sampling time point one, 92 OTUs were identified by MaAsLin2, from which 52 OTUs were positively associated with the drug-free program (Supplementary Table 3). At the genus level, nine taxa were significantly enriched with the drug-free program, whereas 10 taxa were significantly reduced with the drug-free program (Table 4). For sampling time point two, 258 OTUs were positively or negatively associated with the rearing programs (Supplementary Table 4). At the genus level, three taxa were significantly increased, whereas 11 taxa were significantly reduced with the judicious antibiotic use (Table 5). According to flocks from barns that reintroduced antibiotics after the 15-months study, *Sporobacter, Ruminococcus* 2, and *Odoribacter* were found to be positively associated, whereas *Lachnospiraceae* unclassified, *Romboutsia* and *Coriobacteriaceae* unclassified were negatively associated (Supplementary Table 5).

**Table 4:** Bacterial members associated with the drug-free program after the 15-months study using MaAsLin2 at the genus level.

**Drug-free program**	**Taxa**	**Coefficient**	**SE**
Positively associated	*Holdemania*	0.0003	0.0001
	*Anaerofilum*	0.0004	0.0001
	*Ruminococcus* 2	0.0007	0.0002
	Proteobacteria unclassified	0.0009	0.0002
	*Enterococcus*	0.0067	0.0022
	*Parasutterella*	0.0111	0.0029
	*Akkermansia*	0.0178	0.0053
	*Odoribacter*	0.0611	0.0158
	*Bacteroides*	0.0669	0.0339
Negatively associated	*Bacteroidales* unclassified	−0.0328	0.0060
	*Lachnospiraceae* unclassified	−0.0243	0.0061
	*Clostridiales* unclassified	−0.0229	0.0072
	Firmicutes unclassified	−0.0186	0.0102
	Subdoligranulum	−0.0183	0.0086
	*Clostridium* IV	−0.0073	0.0040
	*Anaeroplasma*	−0.0038	0.0012
	*Intestinimonas*	−0.0012	0.0006
	*Coriobacteriaceae* unclassified	−0.0007	0.0001
	*Anaerotruncus*	−0.0002	0.0001

*The positively associated genera are significantly more abundant in the drug-free than conventional program. The negatively associated genera are significantly less abundant in these drug-free flocks. Taxonomic assignment at genus level was not possible for unclassified members*.

**Table 5:** Bacterial members associated with the judicious use of antibiotics, for farms A and B, 6 years after the 15-months study using MaAsLin2 at the genus level.

**Judicious use of antibiotics**	**Taxa**	**Coefficient**	**SE**
Positively associated	*Sporobacter*	0.0001	<0.0001
	*Butyricicoccus*	0.0124	0.0034
	*Butyricimonas*	0.0162	0.0010
Negatively associated	*Ruminococcaceae* unclassified	−0.0365	0.0119
	*Blautia*	−0.0086	0.0035
	*Clostridium* IV	−0.0068	0.0020
	*Clostridium* XlVb	−0.0056	0.0022
	*Clostridia* unclassified	−0.0052	0.0011
	*Intestinimonas*	−0.0038	0.0012
	*Romboutsia*	−0.0016	0.0007
	*Anaeroplasma*	−0.0011	0.0003
	*Ruminococcus* 2	−0.0004	0.0001
	*Coriobacteriaceae* unclassified	−0.0003	0.0001
	*Coriobacteriaceae* unclassified	−0.0002	0.0001

*The positively associated genera are significantly more abundant with the judicious than conventional program (including barns that continued and reintroduced antibiotics after the 15-months study). The negatively associated genera are significantly less abundant in flocks using a judicious program. Taxonomic assignment at genus level was not possible for unclassified members*.

## Discussion

This study, conducted on six commercial broiler chicken farms in Québec, highlights the effects of a short-term antibiotic withdrawal and a long-term judicious use strategy, as well as the conventional antibiotic use, on the dynamics of antibiotic resistance genes and on the cecal bacterial community of broilers.

This study illustrated that for commercial broiler chicken farms, moving to a drug-free program over a 15-months period did not significantly reduce the relative abundance and the absolute copy number of many antibiotic resistance-encoding genes found in bird intestinal contents. Notwithstanding the decrease of *intl1* and *sul1* observed in some drug-free flocks that may be due to a higher fitness cost associated with the carriage of these genes (30). This decrease could also be attributed to a decrease in the selective pressure considering that the use of quaternary ammonium compounds and of sulfonamides may have influenced the persistence of class 1 integrons, which can carry both *sul1* and *qac* resistance genes (31). For instance, a Swedish work studied the impacts of the voluntary restriction on the use of trimethoprim-containing drugs over a 2-years period in Kronoberg County. The results showed a marginal effect on the trimethoprim resistance observed for *E. coli* strains from human urinary tract infections (32). The relative ineffectiveness of such an intervention to significantly impact the antibiotic resistance problem could be explained by the co-selection of antibiotic resistance-encoding genes through the use of other antibiotics or to the low fitness cost associated with the carriage of genes encoding trimethoprim resistance in bacteria (32). In environments, such as broiler chicken farms where the intestinal microbiota corresponds to a signature of the environmental bacterial communities, the fitness cost is considered as one of the most important factors guiding the reduction in the frequency of antibiotic resistance bacteria (30, 33). A decrease in the global antibiotic resistance problem at the community level is then predicted to be measurable years after antibiotic restriction (33). However, the acquisition of compensatory mutations improving fitness for bacteria can jeopardize the reversibility of antimicrobial resistance (30). Despite the lack of evidence on the rate at which resistant bacteria increase or decrease, according to the results of the current study, it could be hypothesized that stopping antibiotics at the farm level over a 15-months period is too short to observe a significant decrease in the abundance of antibiotic resistance genes in the complex ecosystems that are poultry barns for the evaluated genes. The low fitness cost of carrying resistance determinants, the occurrence of compensatory mutations in these bacterial communities, or the use of compounds co-selecting for some resistance determinants probably acted as main drivers. Also, the fact that participating farms of the current study followed the guidelines of the on-farm food safety program of the Chicken Farmers of Canada requiring that farmers wash and disinfect the barn only once a year might have influenced the dynamics of the measured resistance genes on those farms (34). It could also be hypothesized that the use of antibiotics, such as spectinomycin–lincomycin at the hatchery level during the 15-months study period could have contributed to the persistence of some genetic determinants encoding resistance to sulfonamides that are harbored on mobile genetic elements along with *aadA*, a spectinomycin resistance gene (35).

Consistent with previous studies (36, 37), birds submitted to either a conventional or to a drug-free program over a 15-months period did not show major differences for the alpha diversity. Only the inverse Simpson alpha diversity index was marginally increased in conventional flocks, illustrating the stability of the cecal microbiota (38). In contrast, alpha diversity analyses showed interesting changes when comparing flocks from barns using a long-term judicious program with a long-term conventional program (including continued or reintroduced) at sampling time point two. As shown in Table 2, the alpha diversity of birds from flocks raised using a conventional program during and after the 15-months study period was greater than the one observed for birds sampled from flocks that reintroduced antibiotics or adopted a judicious program after the 15-months study period. These observations are in agreement with a previous study where the use of bacitracin increased the richness and the evenness of the chicken cecal microbiota by reducing dominant microorganisms, such as *Lactobacillus* (39). However, no association between *Lactobacillus* and rearing program was documented in the present study. It could be hypothesized that the long-term use of a wide variety of antibiotics, as well as the rotation of these compounds in time, could have depleted some sensitive microorganisms, which in turn could have promoted the growth of other microorganisms. At sampling time point two, it is worth mentioning that no change in the alpha diversity was detected between flocks from barns that had adopted a judicious antibiotic use program and those that had reintroduced antibiotics after the 15-months study. It could be hypothesized that using a conventional program during a longer period of time would have allowed for the cecal microbiota to diversify further in flocks where antibiotics were reintroduced after the 15-months study.

For the cecal community structure, the beta diversity between conventional and drug-free flocks at sampling time point one was significantly different. In addition, at sampling time point two, the beta diversity was significantly different between flocks sampled from barns that adopted a long-term strategy for judiciously using antibiotics, those that continued the conventional rearing program, and those that reintroduced antibiotics after a short-term antibiotic withdrawal. These results are not surprising considering the previous observations of the antibiotic treatment effects on the bacterial community composition of the chicken cecum (39, 40). Results illustrated that a short-term antibiotic withdrawal and a long-term judicious use strategy mainly negatively affected *Ruminococcaceae* and *Lachnospiraceae*, which are the two main families forming the cecal microbiota (41). *Ruminococcaceae* and *Lachnospiraceae* members have the ability to ferment and digest carbohydrates and produce small-chain fatty acids, such as butyrate (41, 42). In a previous work, among 16 butyrate producers from the *Firmicutes*, the clostridial clusters IV and XIVa were associated with the largest production of butyrate (43). Butyrate is an important source of energy for the intestinal epithelium and helps maintain its barrier function by regulating the proliferation of enterocytes (44). In addition to having a negative effect on the colonization of *C. perfringens* (45), it was also found that butyrate enhances performances as evidenced by an increased body weight (46). These last findings were associated with a decrease in *Lactobacillus* and an increase in the ratio of villus height to crypt depth (46). For both sampling time points, antibiotic restriction significantly decreased *Clostridium* IV and *Intestinimonas*. The *Clostridium* cluster IV members includes *Clostridium, Eubacterium, Ruminococcus*, and *Anaerofilum* genera (47). These results are consistent with previous research work in which *Clostridium* IV members were enriched by the use of antimicrobial growth promoters (40). With regard to *Lachnospiraceae*, unclassified members at sampling time point one and *Blautia, Clostridium* XlVb, and *Ruminococcus* 2 at sampling time point two were significantly decreased with both a short-term antibiotic withdrawal and a long-term judicious use strategy. Thus, these members were more abundant in birds raised with a conventional program. These results are in agreement with those of Costa et al., who also observed that *Clostridium* XlVb was significantly enriched with enramycin, a polypeptide antibiotic used at growth-promoting doses (37). In addition, both short-term antibiotic withdrawal and long-term judicious use strategy were significantly associated with a decrease in *Anaeroplasma*, a member of the cecal microbiota for which the role remains unclear (42, 48). Overall, these results suggest that restricting the use of antibiotics tends to decrease the abundance of bacterial populations producing butyrate, which could then affect bird performances and *C. perfringens* colonization. These assumptions are consistent with the results obtained during the previous 15-months study. Indeed, this previous study showed that raising commercial broiler chickens using a drug-free program was negatively impacting the production performance and significantly increasing the occurrence of necrotic enteritis (10). However, due to the low number of farms within each antibiotic use program, the current study did not try to correlate production performances to the microbiota composition at sampling time point two.

Findings of the current study showed significant changes in the abundance of many antibiotic resistance genes depending on both the rearing program and the sampling time point (Figures 2, 3). For farms A and B, which adopted a long-term judicious antibiotic use strategy, a marked decrease in the abundance of various antibiotic resistance genes was observed, whereas this abundance increased for farms using antibiotics on a long-term basis. While minor changes, such as a decrease in *intl1* and *sul1* in some drug-free flocks were observed between barns using either a drug-free or a conventional program after the 15-months study, the 6-years period markedly influenced the abundance of many resistance genes, as predicted by Levin, who examined results of studies that used mathematical models to estimate the time needed for bacterial communities to show reversibility in their antibiotic resistance profile (33). Despite the fact that only two farms adopted a program for judiciously using antibiotics, results from these farms showed a potential impact of addressing the problem of antibiotic resistance by reducing the large-scale use of these compounds, as demonstrated by a systematic review and meta-analysis (49). In contrast, the long-term and routine use of antibiotics on four farms of the current study correlated with a global increase in the abundance of antibiotic resistance-encoding genes. These observations, believed to be influenced by the antibiotic selection pressure, are in agreement with some observations made at the bacterial strain level where the use of some antibiotics was associated with an increase in the prevalence of resistant bacteria to these antibiotics or other antibiotics by co-selection (31, 35). For example, since the voluntary ban on ceftiofur imposed by the poultry industry in Canada in 2014, a mix of spectinomycin and lincomycin was used at the hatchery level to prevent infectious diseases in chicks during the first few days of life (35). It has been reported that the co-selection and selection pressure generated by the preventive use of these two antibiotics at the hatchery level could have selected for gentamicin resistance (35). In addition, the genetic linkage between *vat*(E) and *erm*(B), previously identified in *E. feacium* from European poultry isolates could have contributed to the co-selection of these genes as these appear to be part of a same transposon (50, 51). Similarly, the use of quaternary ammonium compounds as sanitizers in poultry barns and of sulfonamides for the treatment of bacterial infections in commercial broilers could have contributed to the spread of class 1 integrons and could explain the significant increase in the abundance of both *intl1* and *sul1* in farms B, C, D, and F of the current study (Figure 3). Indeed, class 1 integrons can carry both *sul1* and *qac* genes, in addition to being able to capture other resistance genes, such as *aadA* (31, 35).

When comparing sampling time points one and two, a closer examination of the relative abundance and the absolute copy numbers of the targeted genes revealed that the abundance of *vat*(E) decreased markedly in farms A and B, an observation that was not made for the other genes measured. Encoding for an acetyltransferase resulting in streptogramin A resistance in the carrying microorganism, *vat*(E) is found in *Enterococcus feacium*, a microorganism that is part of the intestinal microbiota of broiler chickens (13, 52). As the *vat*(E) gene has been found on transferable plasmids (52), a great negative fitness cost associated with the carriage of this gene by a microorganism or a decrease in the rate of horizontal transfer for this mobile genetic element could both explain the decrease in the abundance of this gene observed on farms reducing the use of antibiotics. However, the rate of horizontal transfer of genes is difficult to predict, just as trends in horizontal antibiotic gene transfer according to the antibiotic exposure levels (53). For farm B (Figure 3), results showed an increase in genes associated with bacitracin and sulfonamide resistances. Considering that this farm had adopted a judicious antibiotic program for several years and that the restriction of the preventive use of antibiotics is a main predisposing factor for the occurrence of necrotic enteritis and of other concomitant bacterial infections (10), these results could reflect an increase in the therapeutic use of these compounds for the treatment of diseased commercial broilers (3). When comparing flocks between sampling time points one and two, results showed a significant increase in the abundance of five to seven targeted genes for farm D, and we could presume that this increasing trend would be attributed to the antibiotic regimen used on this farm over the past 6 years. In addition, the abundance of the *bcrA* and *bcrB* genes increased or decreased jointly in different farms (Figures 2, 3). These observations can be explained by the fact that these two genes are found on the *bcrABDR* operon (29).

This study illustrated an increase in the richness in the samples between points one and two, as well as a marked dispersion of the samples on the NMDS, which was probably attributed to some changes in farm management practices after completion of the 15-months study. Indeed, as all participating farms that adopted a standardized protocol for chick, feed, water, and litter supply, and for coccidiosis management during the 15-months study went back to their previously highly diverse management practices once completing the study, this probably contributed to the changes observed in the cecal microbiota of broiler chickens. As previously described, many farm management factors can influence bird gut microbiota between flocks (54). According to the findings of the present study, it could be hypothesized that a standardization of farm management practices through a common rearing program could normalize the cecum bacterial community composition.

Results pertaining to the detection of the *C. perfringens* alpha toxin-encoding gene were quite unexpected since only 17% of the samples were found positive for the presence of *plc*. In healthy broiler chickens, since the cecum is the main colonization site for *C. perfringens* and because the alpha toxin gene is recognized as a hallmark of all *C. perfringens*, a 100% positivity rate was anticipated (55, 56). This low prevalence suggests that the number of *C. perfringens* in the samples screened was below the previously reported detection limit of 10^3^ bacteria per gram of fecal content (56). Failure to detect the *cpe* gene can therefore be explained since only between 1 and 5% of *C. perfringens* population is known to be enterotoxigenic (57). Interestingly, as the *C. perfringens* population increases during a necrotic enteritis outbreak (55), more than half of the positive samples for the presence of the *plc* gene were identified from flocks experimenting short-term antibiotic withdrawal and long-term judicious use strategy that are recognized to increase the risk of occurrence for this disease.

In conclusion, results from the current study showed that moving to a drug-free program over a 15-months period does not seem to be sufficient to reduce the abundance of many antibiotic resistance-encoding genes, while the judicious use of antibiotics over many years seems to do so. The short-term antibiotic withdrawal and the long-term judicious use strategy changed the bird intestinal microbiota composition, where *Ruminococcaceae* and *Lachnospiraceae* families were negatively impacted, which could be correlated with negative performances and the increase in *C. perfringens* populations. Results also illustrated that adopting a conventional rearing program on commercial broiler chicken farms selected specific antibiotic resistance-encoding genes in many barns. This study highlights the potential impacts of different rearing programs in poultry production and will help develop future policies by guiding science-based decisions on how the use of antibiotics in broiler chicken production should be reduced while maintaining production performance. Reducing antibiotics and using them solely as a therapeutic option could help preserve the effectiveness of these precious tools by contributing to curb the global antibiotic resistance problem.

## Data Availability Statement

The datasets presented in this study can be found in online repositories. The names of the repository/repositories and accession number(s) can be found at: https://www.ncbi.nlm.nih.gov/, PRJNA627503.

## Ethics Statement

The animal study was reviewed and approved by Comité d'Éthique sur l'Utilisation des Animaux (CÉUA) of the Faculté de Médecine Vétérinaire of the Université de Montréal. Written informed consent was obtained from the owners for the participation of their animals in this study.

## Author Contributions

M-LG and AT elaborated the study design. CT, M-LG, and AT made the experiments. GB performed the statistical analyses. All authors analyzed the results, wrote this manuscript, and approved the publication of this work.

## Conflict of Interest

The authors declare that the research was conducted in the absence of any commercial or financial relationships that could be construed as a potential conflict of interest.
