# Supplementary material for: Impacts of Short-Term Antibiotic Withdrawal and Long-Term Judicious Antibiotic Use on Resistance Gene Abundance and Cecal Microbiota Composition on Commercial Broiler Chicken Farms in Québec
Source: Front Vet Sci. 2020 Dec 21;7:547181. doi: 10.3389/fvets.2020.547181 (PMC7779680; doi:10.3389/fvets.2020.547181)
Supplement: Supplementary file 1 [file Data_Sheet_1.docx]

Supplementary Material

Figure S1. Study design A) During the 15-month study, for each participating farm, one barn was on a drug-free program and one barn was on a conventional program. B) After the 15-month study, farms C, D, E and F reintroduced a conventional program in their drug-free barn, while the control barn on those farms was kept on a conventional program. Farms A and B moved to a program for responsibly using antibiotics in both rearing facilities.


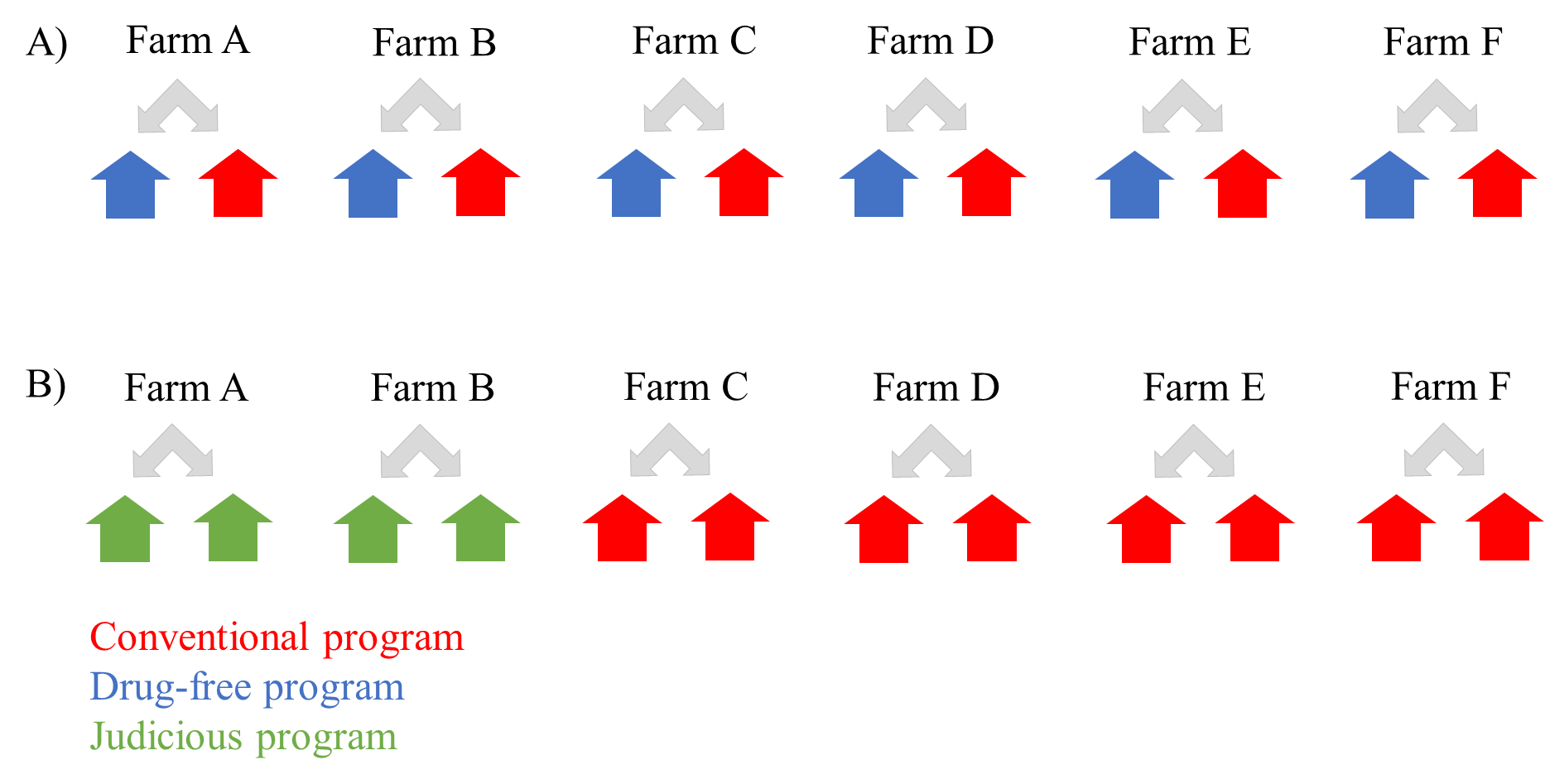


Table S1. Primers and reaction conditions for gene targets PCR amplification.

| Gene | Primer name | Primer sequence (5’–3’) | Amplicon size (bp) | Cycling conditions | 25ul reaction mixture | References |
| --- | --- | --- | --- | --- | --- | --- |
| *bcrA* | *bcrA*_CP-F  *bcrA*_CP-R | GGCAATACCAAGCCGTTGCTTCAT  TTACGAAGCGATACGGAACAGCCA | 408 | 1 cycle of 5 min at 94°C; 30 cycles of 1 min at 94°C, 1 min at 55°C and 1 min at 72°C; 1 cycle of 5 min at 72°C | 5µl of DNA was added to 2.5 µl of 10 x PCR buffer, 0.2 mM of dNTPs, 1.5 mM MgSO4, 320 nM of each primer and 1 U of Taq DNA polymerase | (1) |
| *bcrB* | *bcrB*_CP-F  *bcrB*_CP-R | ATAGGTGGCTGTCCACGGATACAA  CGTTTGTGGGCTATATGACGCTGT | 247 |  |  |  |
| *bcrR* | *bcrR*_CP-F  *bcrR*_CP-R | AAGGGCGGTTACATAGGGTTGTCT  ATGTCTGCTATCGGGCGAAGAACT | 379 |  |  |  |
| *cpe* | GAP11  GAP12 | GGTTCATTAATTGAAACTGGTG  AACGCCAATCATATAAATTACAGC | 154 | 1 cycle of 5 min at 95°C; 45 cycles of 30 sec at 94°C, 30 sec at 55°C, and 30 sec at 72°C; 1 cycle of 5 min at 72°C | 3 µl of DNA was added to 2.5 µl of 10 x PCR buffer, 0.2 mM of dNTPs, 2 mM MgSO_4_, 200 nM of each primer and 1 U of Taq DNA polymerase | (2) |
| *erm*(B) | ermB-F  ermB-R | AAAACTTACCCGCCATACCA  TTTGGCGTGTTTCATTGCTT | 139 | 1 cycle of 5 min at 94°C; 30 cycles of 30 sec at 94°C, 30 sec at 60°C, and 30 sec at 72°C;1 cycle of 5 min at 72°C | 2µl of DNA was added to 2.5 ml of 10 x PCR buffer, 0.2 mM of dNTPs, 1.5 mM MgSO4, 400 nM of each primer and 1 U of Taq DNA polymerase | (3, 4) |
| *intl1* | F6  R6 | GCATCCTCGGTTTTCTGG  GGTGTGGCGGGCTTCGTG | 457 |  |  | (3, 5) |
| *lnu*(B) | LINB1  LINB2 | CCTACCTATTGTTTGTGGAA  ATAACGTTACTCTCCTATTC | 945 | 1 cycle of 5 min at 94°C; 35 cycles of 45 sec at 94°C, 45 sec at 54°C, and 1 min at 72°C; 1 cycle of 5 min at 72°C | 5µl of DNA was added to 2.5 µl of 10 x PCR buffer, 0.2 mM of dNTPs, 2 mM MgSO_4_, 150 nM of each primer and 1.25 U of Taq DNA polymerase | (6, 7) |
| *mcr-1* | CLR5-F  CLR5-R | CGGTCAGTCCGTTTGTTC  CTTGGTCGGTCTGTAGGG | 309 | 1 cycle of 10 min at 95°C; 25 cycles of 10 sec at 95°C, 30 sec at 57°C, and 30 sec at 72°C; 1 cycle of 5 min at 72°C | 5µl of DNA was added to 2.5 µl of 10 x PCR buffer, 0.2 mM of dNTPs, 1.5 mM MgSO_4_, 400 nM of each primer and 1 U of Taq DNA polymerase | (8)  This study |
| *plc* | Cper-plc508-F  Cper-plc508-R | CCGTTGATAGCGCAGGACA  CCCAACTATGACTCATGCTAGCA | 219 | 1 cycle of 5 min at 95°C; 45 cycles of 30 sec at 94°C, 30 sec at 60°C, and 30 sec at 72°C; 1 cycle of 5 min at 72°C | 5µl of DNA was added to 2.5 µl of 10 x PCR buffer, 0.2 mM of dNTPs, 2 mM MgSO_4_, 200 nM of each primer and 1 U of Taq DNA polymerase | (2) |
| *sul1* | F  R | TTCGGCATTCTGAATCTCAC  ATGATCTAACCCTCGGTCTC | 822 | 1 cycle of 5 min at 94°C; 35 cycles of 60 sec at 94°C, 60 sec at 53°C, and 60 sec at 72°C; 1 cycle of 5 min at 72°C | 5µl of DNA was added to 2.5 µl of 10 x PCR buffer, 0.2 mM of dNTPs, 2 mM MgSO_4_, 200 nM of each primer and 1 U of Taq DNA polymerase | (3, 9) |
| *vat*(D) | satA-1  satA-2 | GCTCAATAGGACCAGGTGTA TCCAGCTAACATGTATGGCG | 272 | 1 cycle of 3 min at 94°C; 35 cycles of 1 min at 94°C, 1 min at 55°C, and 1 min at 72°C; 1 cycle of 10 min at 72°C | See *lnu*(B) gene | (7, 10) |
| *vat*(E) | satG-1  satG-2 | ACTATACCTGACGCAAATGC  GGTTCAAATCTTGGTCCG | 512 | 1 cycle of 5 min at 94°C; 30 cycles of 25 sec at 94°C, 40 sec at 52°C, and 50 sec at 72°C; 1 cycle of 6 min at 72°C | See *lnu*(B) gene | (7, 10) |
| *vga*(A) | vga-1  vga-2 | AGTGGTGGTGAAGTAACACG  CTTGTCTCCTCCGCGAATAC | 660 | See *vat*(D) gene | See  *lnu*(B) gene | (10) |
| *vgb*(A) | vgb-1  vgb-2 | TACAGAGTACCCACTACCGA  TCAATTCCTGCTCCAGCAGT | 570 | 1 cycle of 3 min at 94°C; 35 cycles of 1 min at 94°C, 1 min at 52°C, and 1 min at 72°C; 1 cycle of 10 min at 72°C | See *lnu*(B) gene | (10) |

Table S2. Primers, probes and conditions for qPCR amplifications.

| - Gene target | - Primer name | - Primer sequence (5’–3’) | - Amplicon size (bp) | - Cycling conditions | - References |
| --- | --- | --- | --- | --- | --- |
| Universal bacteria,   - *rrnS* | - BACT1369F - PROK1492R - TM1389F | CGGTGAATACGTTCYCGG  GGWTACCTTGTTACGACTT  HEX-CTTGTACACACCGCCCGTC-BHQ1 | - 123 | (Bio-Rad CFX96)  1 cycle of 10 min at 95°C; 40 cycles of 15s at 95°C and 35s at 59°C | - (3, 11) |
| - *bcrA* | - *bcrA*_CP-F - *bcrA*_CP-R | - GGCAATACCAAGCCGTTGCTTCAT - TTACGAAGCGATACGGAACAGCCA | - 408 | - (Roche LC96) - 1 cycle of 10 min at 95°C; 40 cycles of 15s at 95°C and 60s at 61°C; 1 cycle of 10s at 95°C, 60s at 65°C and 1s at 97°C | - (1) |
| - *bcrB* | - *bcrB*_CP-F - *bcrB*_CP-R | - ATAGGTGGCTGTCCACGGATACAA - CGTTTGTGGGCTATATGACGCTGT | - 247 | - (Roche LC96) - 1 cycle of 10 min at 95°C; 40 cycles of 15s at 95°C and 60s at 63°C; 1 cycle of 10s at 95°C, 60s at 65°C and 1s at 97°C | - (1) |
| - *erm*(B) | - ermB-F - ermB-R | - AAAACTTACCCGCCATACCA - TTTGGCGTGTTTCATTGCTT | - 139 | (Bio-Rad CFX96)   - 1 cycle of 10 min at 95°C; 40 cycles of 15s at 95°C and 60s at 65°C; 65°C to 95°C increment of 1°C for 5s | - (3, 4) |
| - *intl1* | IntI1-F2  Intl1-R2 | TCGTGCGTCGCCATCACA  GCTTGTTCTACGGCACGTTTGA | - 67 | (Bio-Rad CFX96)   - 1 cycle of 10 min at 95°C; 40 cycles of 15s at 95°C and 60s at 62°C; 65°C to 95°C increment of 1°C for 5s | (3, 12) |
| - *lnu*(B) | - LINB1 - LINB2 | - CCTACCTATTGTTTGTGGAA - ATAACGTTACTCTCCTATTC | - 945 | - (Roche LC96) - 1 cycle of 10 min at 95°C; 40 cycles of 15s at 95°C and 60s at 58°C; 1 cycle of 10s at 95°C, 60s at 65°C and 1s at 97°C | - (6) |
| - *sul1* | - sul1-F - sul1-R | - GACTGCAGGCTGGTGGTTAT - GAAGAACCGCACAATCTCGT | - 105 | (Bio-Rad CFX96)   - 1 cycle of 10 min at 95°C; 40 cycles of 15s at 95°C and 60s at 64°C; 65°C to 95°C increment of 1°C for 5s | - (3, 13) |
| - *vat*(E) | - satG-1 - satG-2 | - ACTATACCTGACGCAAATGC - GGTTCAAATCTTGGTCCG | - 512 | - (Roche LC96) - 1 cycle of 10 min at 95°C; 40 cycles of 15s at 95°C and 60s at 60°C; 1 cycle of 10s at 95°C, 60s at 65°C and 1s at 97°C | - (10) |

Figure S2. Beta diversity calculated with Bray-Curtis index using ADONIS test with a significance level of 0.05. All comparison were statistically significant (p <.0001). Each point represented one bird sampled. A) Differences between conventional and drug-free programs at sampling time point one. B) Difference between flocks, at sampling time point two, from barns that adopted a long-term judicious use strategy, barns that continued the conventional rearing program and barns that reintroduced antibiotics after a short-term antibiotic withdrawal. C) Differences between sampling time points one and two.


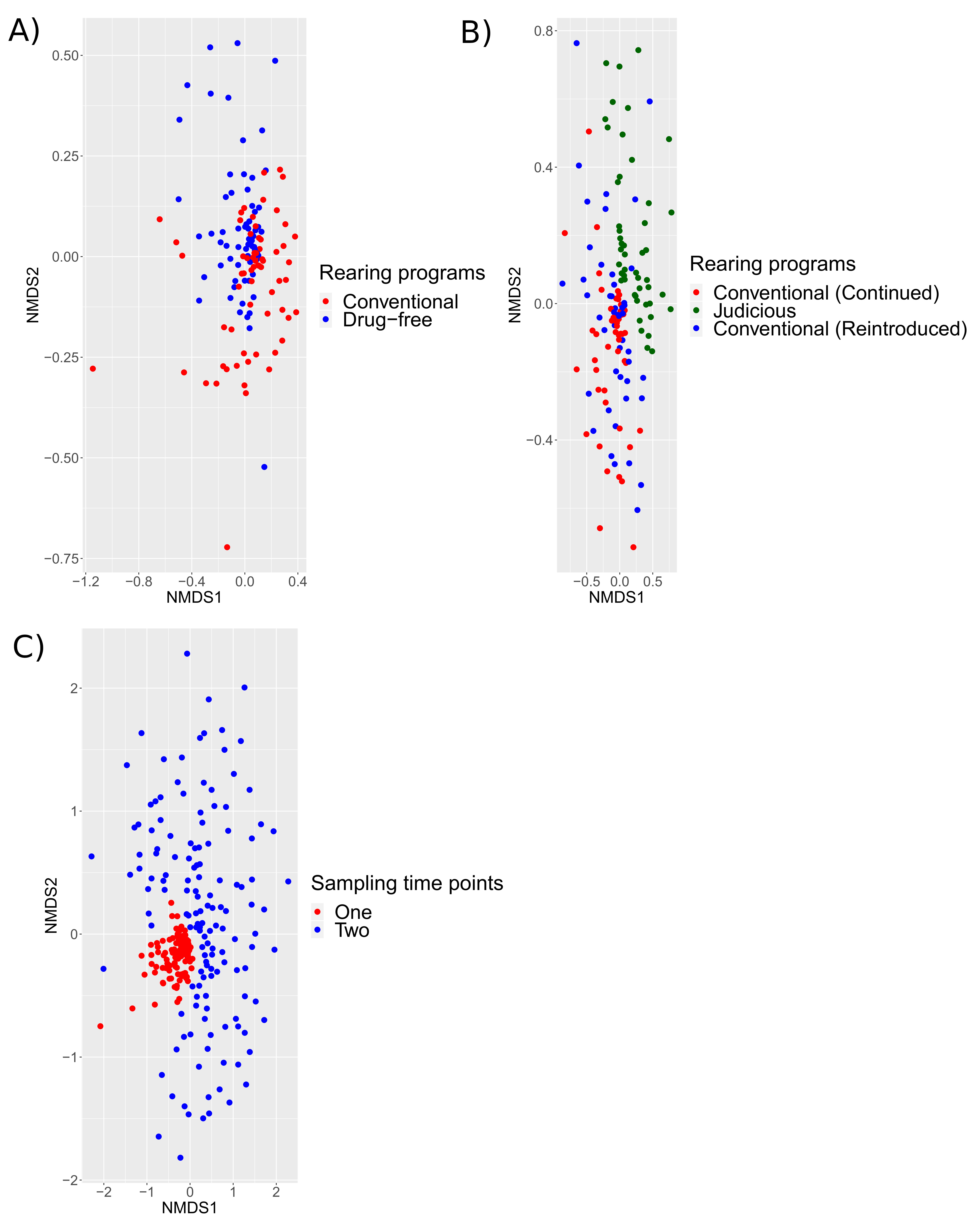


Table S3. Bacterial members associated with the drug-free program after the 15-month study using MaAsLin2 at the OTU level. The positively associated genera are significantly more abundant in the drug-free than conventional program. The negatively associated genera are significantly less abundant in drug-free flocks.

| Taxa | Coefficient | SE |
| --- | --- | --- |
| B,Bacteroidetes,Bacteroidia,Bacteroidales,Bacteroidales_uncl,Bacteroidales_uncl,Otu00007 | -0,032752 | 0,005955 |
| B,Firmicutes,Clostridia,Clostridiales,Ruminococcaceae,Subdoligranulum,Otu00013 | -0,015128 | 0,007514 |
| B,Firmicutes,Clostridia,Clostridiales,Ruminococcaceae,Ruminococcaceae_uncl,Otu00084 | -0,008660 | 0,004099 |
| B,Firmicutes,Firmicutes_uncl,Firmicutes_uncl,Firmicutes_uncl,Firmicutes_uncl,Otu00053 | -0,006701 | 0,003338 |
| B,Firmicutes,Clostridia,Clostridiales,Ruminococcaceae,Butyricicoccus,Otu00080 | -0,006242 | 0,001488 |
| B,Firmicutes,Clostridia,Clostridiales,Clostridiales_uncl,Clostridiales_uncl,Otu00101 | -0,004496 | 0,000921 |
| B,Firmicutes,Clostridia,Clostridiales,Lachnospiraceae,Clostridium_XlVa,Otu00061 | -0,003935 | 0,001483 |
| B,Tenericutes,Mollicutes,Anaeroplasmatales,Anaeroplasmataceae,Anaeroplasma,Otu00122 | -0,003772 | 0,001187 |
| B,Firmicutes,Clostridia,Clostridiales,Clostridiales_uncl,Clostridiales_uncl,Otu00067 | -0,003727 | 0,001554 |
| B,Firmicutes,Clostridia,Clostridiales,Lachnospiraceae,Lachnospiraceae_uncl,Otu00056 | -0,003103 | 0,001523 |
| B,Firmicutes,Clostridia,Clostridiales,Clostridiales_uncl,Clostridiales_uncl,Otu00152 | -0,002807 | 0,000650 |
| B,B_uncl,B_uncl,B_uncl,B_uncl,B_uncl,Otu00077 | -0,002122 | 0,000674 |
| B,Firmicutes,Clostridia,Clostridiales,Lachnospiraceae,Lachnospiraceae_uncl,Otu00117 | -0,002057 | 0,000653 |
| B,Firmicutes,Clostridia,Clostridiales,Clostridiales_uncl,Clostridiales_uncl,Otu00208 | -0,001862 | 0,000239 |
| B,Firmicutes,Firmicutes_uncl,Firmicutes_uncl,Firmicutes_uncl,Firmicutes_uncl,Otu00205 | -0,001400 | 0,000197 |
| B,Firmicutes,Clostridia,Clostridiales,Ruminococcaceae,Ruminococcaceae_uncl,Otu00200 | -0,001398 | 0,000419 |
| B,Firmicutes,Firmicutes_uncl,Firmicutes_uncl,Firmicutes_uncl,Firmicutes_uncl,Otu00222 | -0,001396 | 0,000270 |
| B,Firmicutes,Clostridia,Clostridiales,Lachnospiraceae,Lachnospiraceae_uncl,Otu00243 | -0,001393 | 0,000438 |
| B,Firmicutes,Clostridia,Clostridiales,Ruminococcaceae,Oscillibacter,Otu00106 | -0,001383 | 0,000520 |
| B,Firmicutes,Clostridia,Clostridiales,Clostridiales_uncl,Clostridiales_uncl,Otu00250 | -0,001283 | 0,000151 |
| B,B_uncl,B_uncl,B_uncl,B_uncl,B_uncl,Otu00133 | -0,001166 | 0,000446 |
| B,Firmicutes,Clostridia,Clostridiales,Lachnospiraceae,Lachnospiraceae_uncl,Otu00288 | -0,001134 | 0,000371 |
| B,Firmicutes,Clostridia,Clostridiales,Ruminococcaceae,Ruminococcus,Otu00187 | -0,001064 | 0,000268 |
| B,Firmicutes,Erysipelotrichia,Erysipelotrichales,Erysipelotrichaceae,Erysipelotrichaceae_uncl,Otu00043 | -0,001027 | 0,000363 |
| B,Firmicutes,Clostridia,Clostridiales,Clostridiales_uncl,Clostridiales_uncl,Otu00271 | -0,000863 | 0,000249 |
| B,Firmicutes,Clostridia,Clostridiales,Lachnospiraceae,Lachnospiraceae_uncl,Otu00214 | -0,000830 | 0,000339 |
| B,Firmicutes,Clostridia,Clostridiales,Clostridiales_uncl,Clostridiales_uncl,Otu00167 | -0,000780 | 0,000327 |
| B,B_uncl,B_uncl,B_uncl,B_uncl,B_uncl,Otu00254 | -0,000736 | 0,000183 |
| B,Firmicutes,Clostridia,Clostridiales,Lachnospiraceae,Lachnospiraceae_uncl,Otu00209 | -0,000688 | 0,000291 |
| B,Firmicutes,Clostridia,Clostridiales,Ruminococcaceae,Ruminococcaceae_uncl,Otu00272 | -0,000547 | 0,000146 |
| B,Firmicutes,Clostridia,Clostridiales,Clostridiales_uncl,Clostridiales_uncl,Otu00213 | -0,000528 | 0,000269 |
| B,Firmicutes,Clostridia,Clostridiales,Ruminococcaceae,Clostridium_IV,Otu00352 | -0,000407 | 0,000075 |
| B,Firmicutes,Clostridia,Clostridiales,Ruminococcaceae,Ruminococcaceae_uncl,Otu00422 | -0,000371 | 0,000057 |
| B,ActinoB,ActinoB,CorioBles,CorioBceae,CorioBceae_uncl,Otu00386 | -0,000365 | 0,000066 |
| B,Firmicutes,Clostridia,Clostridiales,Ruminococcaceae,Clostridium_IV,Otu00404 | -0,000334 | 0,000066 |
| B,Firmicutes,Clostridia,Clostridiales,Lachnospiraceae,Lachnospiraceae_uncl,Otu00380 | -0,000260 | 0,000076 |
| B,ActinoB,ActinoB,CorioBles,CorioBceae,CorioBceae_uncl,Otu00466 | -0,000258 | 0,000059 |
| B,Firmicutes,Clostridia,Clostridiales,Lachnospiraceae,Lachnospiraceae_uncl,Otu00392 | -0,000253 | 0,000076 |
| B,Firmicutes,Clostridia,Clostridiales,Clostridiales_uncl,Clostridiales_uncl,Otu00451 | -0,000251 | 0,000049 |
| B,Firmicutes,Clostridia,Clostridiales,Ruminococcaceae,Anaerotruncus,Otu00312 | -0,000241 | 0,000096 |
| B,Firmicutes,Clostridia,Clostridiales,Lachnospiraceae,Lachnospiraceae_uncl,Otu00507 | -0,000177 | 0,000018 |
| B,Firmicutes,Clostridia,Clostridiales,Clostridiales_uncl,Clostridiales_uncl,Otu00568 | -0,000176 | 0,000047 |
| B,Firmicutes,Clostridia,Clostridiales,Ruminococcaceae,Ruminococcaceae_uncl,Otu00342 | -0,000133 | 0,000063 |
| B,Firmicutes,Clostridia,Clostridiales,Ruminococcaceae,Ruminococcaceae_uncl,Otu00607 | 0,000113 | 0,000019 |
| B,Firmicutes,Clostridia,Clostridia_uncl,Clostridia_uncl,Clostridia_uncl,Otu00589 | 0,000125 | 0,000014 |
| B,Firmicutes,Clostridia,Clostridiales,Clostridiales_uncl,Clostridiales_uncl,Otu00585 | 0,000128 | 0,000014 |
| B,Firmicutes,Clostridia,Clostridiales,Ruminococcaceae,Ruminococcaceae_uncl,Otu00539 | 0,000183 | 0,000045 |
| B,Firmicutes,Clostridia,Clostridiales,Ruminococcaceae,Clostridium_IV,Otu00448 | 0,000185 | 0,000050 |
| B,Firmicutes,Clostridia,Clostridiales,Ruminococcaceae,Ruminococcaceae_uncl,Otu00526 | 0,000215 | 0,000023 |
| B,Firmicutes,Clostridia,Clostridiales,Ruminococcaceae,Ruminococcaceae_uncl,Otu00408 | 0,000250 | 0,000069 |
| B,Firmicutes,Erysipelotrichia,Erysipelotrichales,Erysipelotrichaceae,Holdemania,Otu00433 | 0,000252 | 0,000054 |
| B,Firmicutes,Clostridia,Clostridiales,Ruminococcaceae,Clostridium_III,Otu00435 | 0,000276 | 0,000049 |
| B,Firmicutes,Clostridia,Clostridiales,Ruminococcaceae,Ruminococcaceae_uncl,Otu00424 | 0,000276 | 0,000074 |
| B,Firmicutes,Clostridia,Clostridiales,Ruminococcaceae,Ruminococcaceae_uncl,Otu00361 | 0,000290 | 0,000053 |
| B,B_uncl,B_uncl,B_uncl,B_uncl,B_uncl,Otu00427 | 0,000304 | 0,000040 |
| B,Firmicutes,Clostridia,Clostridiales,Ruminococcaceae,Ruminococcaceae_uncl,Otu00349 | 0,000357 | 0,000099 |
| B,Firmicutes,Clostridia,Clostridiales,Ruminococcaceae,Anaerofilum,Otu00321 | 0,000373 | 0,000088 |
| B,Firmicutes,Clostridia,Clostridiales,Ruminococcaceae,Ruminococcaceae_uncl,Otu00360 | 0,000391 | 0,000179 |
| B,B_uncl,B_uncl,B_uncl,B_uncl,B_uncl,Otu00362 | 0,000420 | 0,000095 |
| B,Firmicutes,Clostridia,Clostridiales,Lachnospiraceae,Lachnospiraceae_uncl,Otu00420 | 0,000427 | 0,000139 |
| B,B_uncl,B_uncl,B_uncl,B_uncl,B_uncl,Otu00226 | 0,000431 | 0,000092 |
| B,B_uncl,B_uncl,B_uncl,B_uncl,B_uncl,Otu00327 | 0,000476 | 0,000067 |
| B,Firmicutes,Clostridia,Clostridiales,Clostridiales_uncl,Clostridiales_uncl,Otu00313 | 0,000488 | 0,000093 |
| B,Firmicutes,Clostridia,Clostridiales,Ruminococcaceae,Oscillibacter,Otu00290 | 0,000539 | 0,000071 |
| B,Firmicutes,Erysipelotrichia,Erysipelotrichales,Erysipelotrichaceae,Erysipelotrichaceae_uncl,Otu00102 | 0,000619 | 0,000204 |
| B,Firmicutes,Clostridia,Clostridiales,Ruminococcaceae,Clostridium_IV,Otu00276 | 0,000649 | 0,000189 |
| B,Firmicutes,Clostridia,Clostridiales,Lachnospiraceae,Ruminococcus2,Otu00258 | 0,000662 | 0,000156 |
| B,Firmicutes,Clostridia,Clostridiales,Ruminococcaceae,Ruminococcaceae_uncl,Otu00317 | 0,000744 | 0,000191 |
| B,Firmicutes,Clostridia,Clostridiales,Ruminococcaceae,Ruminococcaceae_uncl,Otu00301 | 0,000828 | 0,000200 |
| B,Firmicutes,Clostridia,Clostridiales,Lachnospiraceae,Lachnospiraceae_uncl,Otu00270 | 0,000864 | 0,000109 |
| B,Firmicutes,Clostridia,Clostridiales,Clostridiales_uncl,Clostridiales_uncl,Otu00268 | 0,000875 | 0,000296 |
| B,ProteoB,ProteoB_uncl,ProteoB_uncl,ProteoB_uncl,ProteoB_uncl,Otu00225 | 0,000923 | 0,000241 |
| B,Firmicutes,Clostridia,Clostridiales,Ruminococcaceae,Pseudoflavonifractor,Otu00201 | 0,000931 | 0,000215 |
| B,B_uncl,B_uncl,B_uncl,B_uncl,B_uncl,Otu00306 | 0,000963 | 0,000322 |
| B,Firmicutes,Firmicutes_uncl,Firmicutes_uncl,Firmicutes_uncl,Firmicutes_uncl,Otu00194 | 0,000997 | 0,000180 |
| B,B_uncl,B_uncl,B_uncl,B_uncl,B_uncl,Otu00256 | 0,001030 | 0,000191 |
| B,Firmicutes,Clostridia,Clostridiales,Clostridiales_uncl,Clostridiales_uncl,Otu00202 | 0,001042 | 0,000144 |
| B,Firmicutes,Clostridia,Clostridiales,Ruminococcaceae,Faecalibacterium,Otu00158 | 0,001048 | 0,000163 |
| B,Firmicutes,Clostridia,Clostridiales,Clostridiales_uncl,Clostridiales_uncl,Otu00216 | 0,001427 | 0,000448 |
| B,Firmicutes,Firmicutes_uncl,Firmicutes_uncl,Firmicutes_uncl,Firmicutes_uncl,Otu00203 | 0,001548 | 0,000404 |
| B,Firmicutes,Clostridia,Clostridiales,Lachnospiraceae,Lachnospiraceae_uncl,Otu00124 | 0,001806 | 0,000739 |
| B,B_uncl,B_uncl,B_uncl,B_uncl,B_uncl,Otu00140 | 0,001839 | 0,000427 |
| B,Firmicutes,Clostridia,Clostridiales,Clostridiales_uncl,Clostridiales_uncl,Otu00112 | 0,002827 | 0,000781 |
| B,Firmicutes,Clostridia,Clostridiales,Ruminococcaceae,Subdoligranulum,Otu00125 | 0,003300 | 0,000670 |
| B,B_uncl,B_uncl,B_uncl,B_uncl,B_uncl,Otu00107 | 0,003433 | 0,000736 |
| B,Firmicutes,Clostridia,Clostridiales,Lachnospiraceae,Clostridium_XlVb,Otu00130 | 0,003580 | 0,000919 |
| B,Firmicutes,Clostridia,Clostridiales,Lachnospiraceae,Blautia,Otu00147 | 0,003638 | 0,000903 |
| B,Firmicutes,Clostridia,Clostridiales,Ruminococcaceae,Ruminococcaceae_uncl,Otu00079 | 0,003996 | 0,001248 |
| B,Firmicutes,Clostridia,Clostridiales,Clostridiales_uncl,Clostridiales_uncl,Otu00088 | 0,005009 | 0,001922 |
| B,Firmicutes,Bacilli,Lactobacillales,Enterococcaceae,Enterococcus,Otu00058 | 0,006746 | 0,002229 |
| B,ProteoB,BetaproteoB,Burkholderiales,Sutterellaceae,Parasutterella,Otu00011 | 0,011086 | 0,002948 |
| B,Verrucomicrobia,Verrucomicrobiae,Verrucomicrobiales,Verrucomicrobiaceae,Akkermansia,Otu00022 | 0,017810 | 0,005260 |
| B,Firmicutes,Clostridia,Clostridiales,Ruminococcaceae,Faecalibacterium,Otu00028 | 0,021493 | 0,007789 |
| B,Bacteroidetes,Bacteroidia,Bacteroidales,Porphyromonadaceae,Odoribacter,Otu00003 | 0,061067 | 0,015814 |
| B,Bacteroidetes,Bacteroidia,Bacteroidales,Bacteroidaceae,Bacteroides,Otu00001 | 0,075074 | 0,030693 |

Table S4. Bacterial members associated with the rearing programs, at sampling time point two, using MaAsLin2 at the OTU level. Negative values are negatively associated to the rearing program indicated and positive values are positively associated to the rearing program indicated.

| taxa | Rearing programs | coefficient | SE |
| --- | --- | --- | --- |
| Bacteria,Firmicutes,Clostridia,Clostridiales,Ruminococcaceae,Ruminococcaceae_unclassified,Otu00025 | Judicious | -0,010362 | 0,001305 |
| Bacteria,Firmicutes,Clostridia,Clostridiales,Ruminococcaceae,Subdoligranulum,Otu00016 | Judicious | -0,008222 | 0,003583 |
| Bacteria,Firmicutes,Clostridia,Clostridiales,Lachnospiraceae,Blautia,Otu00017 | Judicious | -0,007762 | 0,003461 |
| Bacteria,Firmicutes,Clostridia,Clostridiales,Lachnospiraceae,Clostridium_XlVb,Otu00021 | Judicious | -0,006684 | 0,002207 |
| Bacteria,Firmicutes,Firmicutes_unclassified,Firmicutes_unclassified,Firmicutes_unclassified,Firmicutes_unclassified,Otu00029 | Judicious | -0,006198 | 0,002748 |
| Bacteria,Firmicutes,Clostridia,Clostridiales,Ruminococcaceae,Faecalibacterium,Otu00066 | Judicious | -0,005921 | 0,001293 |
| Bacteria,Firmicutes,Clostridia,Clostridiales,Clostridiales_unclassified,Clostridiales_unclassified,Otu00067 | Judicious | -0,005398 | 0,001231 |
| Bacteria,Firmicutes,Clostridia,Clostridiales,Ruminococcaceae,Faecalibacterium,Otu00028 | Judicious | -0,005352 | 0,002506 |
| Bacteria,Firmicutes,Clostridia,Clostridiales,Ruminococcaceae,Ruminococcaceae_unclassified,Otu00035 | Judicious | -0,004929 | 0,001594 |
| Bacteria,Firmicutes,Bacilli,Lactobacillales,Enterococcaceae,Enterococcus,Otu00058 | Reintroduced | -0,004043 | 0,001898 |
| Bacteria,Firmicutes,Clostridia,Clostridiales,Clostridiales_unclassified,Clostridiales_unclassified,Otu00047 | Judicious | -0,003952 | 0,001915 |
| Bacteria,Firmicutes,Clostridia,Clostridiales,Ruminococcaceae,Ruminococcaceae_unclassified,Otu00025 | Reintroduced | -0,003729 | 0,001305 |
| Bacteria,Firmicutes,Clostridia,Clostridiales,Clostridiales_unclassified,Clostridiales_unclassified,Otu00064 | Judicious | -0,003688 | 0,001193 |
| Bacteria,Firmicutes,Clostridia,Clostridiales,Ruminococcaceae,Clostridium_IV,Otu00034 | Judicious | -0,003636 | 0,001093 |
| Bacteria,Firmicutes,Clostridia,Clostridiales,Lachnospiraceae,Lachnospiraceae_unclassified,Otu00032 | Judicious | -0,003555 | 0,001604 |
| Bacteria,Firmicutes,Firmicutes_unclassified,Firmicutes_unclassified,Firmicutes_unclassified,Firmicutes_unclassified,Otu00113 | Judicious | -0,003177 | 0,000813 |
| Bacteria,Firmicutes,Clostridia,Clostridiales,Lachnospiraceae,Lachnospiraceae_unclassified,Otu00042 | Reintroduced | -0,002852 | 0,001121 |
| Bacteria,Firmicutes,Clostridia,Clostridiales,Ruminococcaceae,Faecalibacterium,Otu00066 | Reintroduced | -0,002604 | 0,001293 |
| Bacteria,Bacteria_unclassified,Bacteria_unclassified,Bacteria_unclassified,Bacteria_unclassified,Bacteria_unclassified,Otu00077 | Judicious | -0,002369 | 0,000913 |
| Bacteria,Firmicutes,Clostridia,Clostridiales,Clostridiales_unclassified,Clostridiales_unclassified,Otu00088 | Judicious | -0,002315 | 0,000785 |
| Bacteria,Firmicutes,Clostridia,Clostridiales,Ruminococcaceae,Ruminococcaceae_unclassified,Otu00154 | Judicious | -0,002304 | 0,000564 |
| Bacteria,Bacteria_unclassified,Bacteria_unclassified,Bacteria_unclassified,Bacteria_unclassified,Bacteria_unclassified,Otu00215 | Judicious | -0,002290 | 0,001032 |
| Bacteria,Bacteria_unclassified,Bacteria_unclassified,Bacteria_unclassified,Bacteria_unclassified,Bacteria_unclassified,Otu00162 | Judicious | -0,002229 | 0,000390 |
| Bacteria,Firmicutes,Clostridia,Clostridiales,Lachnospiraceae,Lachnospiraceae_unclassified,Otu00060 | Judicious | -0,002131 | 0,001009 |
| Bacteria,Firmicutes,Clostridia,Clostridia_unclassified,Clostridia_unclassified,Clostridia_unclassified,Otu00192 | Judicious | -0,002114 | 0,000845 |
| Bacteria,Firmicutes,Clostridia,Clostridiales,Clostridiales_unclassified,Clostridiales_unclassified,Otu00131 | Judicious | -0,002085 | 0,000691 |
| Bacteria,Firmicutes,Clostridia,Clostridiales,Peptostreptococcaceae,Romboutsia,Otu00184 | Reintroduced | -0,001924 | 0,000680 |
| Bacteria,Firmicutes,Firmicutes_unclassified,Firmicutes_unclassified,Firmicutes_unclassified,Firmicutes_unclassified,Otu00068 | Reintroduced | -0,001915 | 0,000609 |
| Bacteria,Firmicutes,Clostridia,Clostridiales,Ruminococcaceae,Intestinimonas,Otu00111 | Judicious | -0,001874 | 0,000450 |
| Bacteria,Firmicutes,Clostridia,Clostridiales,Lachnospiraceae,Lachnospiraceae_unclassified,Otu00054 | Judicious | -0,001813 | 0,000512 |
| Bacteria,Firmicutes,Clostridia,Clostridiales,Peptostreptococcaceae,Romboutsia,Otu00184 | Judicious | -0,001589 | 0,000680 |
| Bacteria,Firmicutes,Clostridia,Clostridiales,Lachnospiraceae,Lachnospiraceae_unclassified,Otu00135 | Reintroduced | -0,001527 | 0,000561 |
| Bacteria,Firmicutes,Clostridia,Clostridiales,Lachnospiraceae,Lachnospiraceae_unclassified,Otu00054 | Reintroduced | -0,001489 | 0,000512 |
| Bacteria,Firmicutes,Clostridia,Clostridiales,Lachnospiraceae,Lachnospiraceae_unclassified,Otu00135 | Judicious | -0,001381 | 0,000561 |
| Bacteria,Firmicutes,Clostridia,Clostridiales,Clostridiales_unclassified,Clostridiales_unclassified,Otu00169 | Judicious | -0,001378 | 0,000420 |
| Bacteria,Firmicutes,Firmicutes_unclassified,Firmicutes_unclassified,Firmicutes_unclassified,Firmicutes_unclassified,Otu00199 | Judicious | -0,001302 | 0,000245 |
| Bacteria,Firmicutes,Clostridia,Clostridiales,Ruminococcaceae,Ruminococcaceae_unclassified,Otu00082 | Judicious | -0,001190 | 0,000556 |
| Bacteria,Firmicutes,Clostridia,Clostridia_unclassified,Clostridia_unclassified,Clostridia_unclassified,Otu00190 | Judicious | -0,001148 | 0,000268 |
| Bacteria,Firmicutes,Clostridia,Clostridia_unclassified,Clostridia_unclassified,Clostridia_unclassified,Otu00190 | Reintroduced | -0,001148 | 0,000268 |
| Bacteria,Tenericutes,Mollicutes,Anaeroplasmatales,Anaeroplasmataceae,Anaeroplasma,Otu00122 | Judicious | -0,001140 | 0,000257 |
| Bacteria,Firmicutes,Clostridia,Clostridiales,Ruminococcaceae,Ruminococcaceae_unclassified,Otu00100 | Judicious | -0,001134 | 0,000189 |
| Bacteria,Firmicutes,Clostridia,Clostridiales,Lachnospiraceae,Lachnospiraceae_unclassified,Otu00210 | Judicious | -0,001053 | 0,000354 |
| Bacteria,Firmicutes,Clostridia,Clostridiales,Ruminococcaceae,Ruminococcaceae_unclassified,Otu00195 | Judicious | -0,001019 | 0,000352 |
| Bacteria,Firmicutes,Clostridia,Clostridiales,Ruminococcaceae,Ruminococcaceae_unclassified,Otu00200 | Judicious | -0,000966 | 0,000244 |
| Bacteria,Firmicutes,Clostridia,Clostridiales,Clostridiales_unclassified,Clostridiales_unclassified,Otu00101 | Reintroduced | -0,000947 | 0,000481 |
| Bacteria,Firmicutes,Clostridia,Clostridiales,Clostridiales_unclassified,Clostridiales_unclassified,Otu00160 | Judicious | -0,000930 | 0,000173 |
| Bacteria,Firmicutes,Firmicutes_unclassified,Firmicutes_unclassified,Firmicutes_unclassified,Firmicutes_unclassified,Otu00281 | Judicious | -0,000930 | 0,000305 |
| Bacteria,Firmicutes,Firmicutes_unclassified,Firmicutes_unclassified,Firmicutes_unclassified,Firmicutes_unclassified,Otu00281 | Reintroduced | -0,000930 | 0,000305 |
| Bacteria,Firmicutes,Clostridia,Clostridiales,Ruminococcaceae,Ruminococcaceae_unclassified,Otu00137 | Judicious | -0,000928 | 0,000391 |
| Bacteria,Firmicutes,Clostridia,Clostridiales,Lachnospiraceae,Lachnospiraceae_unclassified,Otu00209 | Judicious | -0,000905 | 0,000377 |
| Bacteria,Firmicutes,Firmicutes_unclassified,Firmicutes_unclassified,Firmicutes_unclassified,Firmicutes_unclassified,Otu00264 | Judicious | -0,000895 | 0,000312 |
| Bacteria,Bacteria_unclassified,Bacteria_unclassified,Bacteria_unclassified,Bacteria_unclassified,Bacteria_unclassified,Otu00161 | Judicious | -0,000846 | 0,000417 |
| Bacteria,Bacteria_unclassified,Bacteria_unclassified,Bacteria_unclassified,Bacteria_unclassified,Bacteria_unclassified,Otu00282 | Judicious | -0,000825 | 0,000239 |
| Bacteria,Bacteria_unclassified,Bacteria_unclassified,Bacteria_unclassified,Bacteria_unclassified,Bacteria_unclassified,Otu00282 | Reintroduced | -0,000825 | 0,000239 |
| Bacteria,Firmicutes,Clostridia,Clostridiales,Clostridiales_unclassified,Clostridiales_unclassified,Otu00231 | Judicious | -0,000763 | 0,000155 |
| Bacteria,Firmicutes,Clostridia,Clostridiales,Clostridiales_unclassified,Clostridiales_unclassified,Otu00231 | Reintroduced | -0,000763 | 0,000155 |
| Bacteria,Firmicutes,Clostridia,Clostridiales,Clostridiales_unclassified,Clostridiales_unclassified,Otu00245 | Judicious | -0,000756 | 0,000116 |
| Bacteria,Firmicutes,Clostridia,Clostridiales,Clostridiales_unclassified,Clostridiales_unclassified,Otu00245 | Reintroduced | -0,000756 | 0,000116 |
| Bacteria,Firmicutes,Clostridia,Clostridiales,Clostridiales_unclassified,Clostridiales_unclassified,Otu00218 | Judicious | -0,000694 | 0,000133 |
| Bacteria,Bacteria_unclassified,Bacteria_unclassified,Bacteria_unclassified,Bacteria_unclassified,Bacteria_unclassified,Otu00327 | Judicious | -0,000679 | 0,000245 |
| Bacteria,Firmicutes,Clostridia,Clostridiales,Clostridiales_unclassified,Clostridiales_unclassified,Otu00208 | Reintroduced | -0,000672 | 0,000216 |
| Bacteria,Bacteria_unclassified,Bacteria_unclassified,Bacteria_unclassified,Bacteria_unclassified,Bacteria_unclassified,Otu00255 | Judicious | -0,000613 | 0,000172 |
| Bacteria,Bacteria_unclassified,Bacteria_unclassified,Bacteria_unclassified,Bacteria_unclassified,Bacteria_unclassified,Otu00255 | Reintroduced | -0,000613 | 0,000172 |
| Bacteria,Firmicutes,Clostridia,Clostridiales,Clostridiales_unclassified,Clostridiales_unclassified,Otu00293 | Judicious | -0,000584 | 0,000175 |
| Bacteria,Firmicutes,Clostridia,Clostridiales,Clostridiales_unclassified,Clostridiales_unclassified,Otu00293 | Reintroduced | -0,000584 | 0,000175 |
| Bacteria,Firmicutes,Clostridia,Clostridiales,Clostridiales_unclassified,Clostridiales_unclassified,Otu00331 | Judicious | -0,000584 | 0,000198 |
| Bacteria,Firmicutes,Clostridia,Clostridiales,Clostridiales_unclassified,Clostridiales_unclassified,Otu00253 | Judicious | -0,000583 | 0,000131 |
| Bacteria,Firmicutes,Clostridia,Clostridiales,Clostridiales_unclassified,Clostridiales_unclassified,Otu00249 | Judicious | -0,000573 | 0,000221 |
| Bacteria,Firmicutes,Clostridia,Clostridiales,Clostridiales_unclassified,Clostridiales_unclassified,Otu00249 | Reintroduced | -0,000573 | 0,000221 |
| Bacteria,Firmicutes,Firmicutes_unclassified,Firmicutes_unclassified,Firmicutes_unclassified,Firmicutes_unclassified,Otu00179 | Judicious | -0,000568 | 0,000299 |
| Bacteria,Firmicutes,Clostridia,Clostridiales,Clostridiales_unclassified,Clostridiales_unclassified,Otu00250 | Judicious | -0,000548 | 0,000157 |
| Bacteria,Firmicutes,Clostridia,Clostridiales,Ruminococcaceae,Oscillibacter,Otu00289 | Judicious | -0,000541 | 0,000156 |
| Bacteria,Firmicutes,Firmicutes_unclassified,Firmicutes_unclassified,Firmicutes_unclassified,Firmicutes_unclassified,Otu00234 | Judicious | -0,000534 | 0,000219 |
| Bacteria,Bacteria_unclassified,Bacteria_unclassified,Bacteria_unclassified,Bacteria_unclassified,Bacteria_unclassified,Otu00291 | Judicious | -0,000529 | 0,000265 |
| Bacteria,Bacteria_unclassified,Bacteria_unclassified,Bacteria_unclassified,Bacteria_unclassified,Bacteria_unclassified,Otu00256 | Judicious | -0,000526 | 0,000252 |
| Bacteria,Firmicutes,Clostridia,Clostridiales,Clostridiales_unclassified,Clostridiales_unclassified,Otu00197 | Judicious | -0,000518 | 0,000107 |
| Bacteria,Firmicutes,Clostridia,Clostridiales,Ruminococcaceae,Ruminococcaceae_unclassified,Otu00100 | Reintroduced | -0,000494 | 0,000189 |
| Bacteria,Firmicutes,Clostridia,Clostridiales,Ruminococcaceae,Clostridium_IV,Otu00276 | Judicious | -0,000483 | 0,000062 |
| Bacteria,Firmicutes,Clostridia,Clostridiales,Clostridiales_unclassified,Clostridiales_unclassified,Otu00241 | Judicious | -0,000459 | 0,000191 |
| Bacteria,Firmicutes,Clostridia,Clostridiales,Clostridiales_unclassified,Clostridiales_unclassified,Otu00246 | Judicious | -0,000455 | 0,000100 |
| Bacteria,Firmicutes,Clostridia,Clostridiales,Clostridiales_unclassified,Clostridiales_unclassified,Otu00246 | Reintroduced | -0,000455 | 0,000100 |
| Bacteria,Firmicutes,Clostridia,Clostridiales,Clostridiales_unclassified,Clostridiales_unclassified,Otu00376 | Judicious | -0,000453 | 0,000156 |
| Bacteria,Firmicutes,Clostridia,Clostridiales,Clostridiales_unclassified,Clostridiales_unclassified,Otu00376 | Reintroduced | -0,000453 | 0,000156 |
| Bacteria,Firmicutes,Clostridia,Clostridiales,Ruminococcaceae,Ruminococcaceae_unclassified,Otu00472 | Judicious | -0,000447 | 0,000094 |
| Bacteria,Firmicutes,Clostridia,Clostridiales,Ruminococcaceae,Oscillibacter,Otu00398 | Judicious | -0,000444 | 0,000069 |
| Bacteria,Actinobacteria,Actinobacteria,Coriobacteriales,Coriobacteriaceae,Gordonibacter,Otu00334 | Judicious | -0,000443 | 0,000094 |
| Bacteria,Firmicutes,Clostridia,Clostridiales,Ruminococcaceae,Ruminococcaceae_unclassified,Otu00375 | Judicious | -0,000437 | 0,000134 |
| Bacteria,Firmicutes,Clostridia,Clostridiales,Ruminococcaceae,Ruminococcaceae_unclassified,Otu00375 | Reintroduced | -0,000437 | 0,000134 |
| Bacteria,Firmicutes,Clostridia,Clostridiales,Lachnospiraceae,Lachnospiraceae_unclassified,Otu00270 | Reintroduced | -0,000426 | 0,000142 |
| Bacteria,Firmicutes,Clostridia,Clostridiales,Ruminococcaceae,Ruminococcaceae_unclassified,Otu00389 | Judicious | -0,000402 | 0,000098 |
| Bacteria,Firmicutes,Clostridia,Clostridiales,Ruminococcaceae,Ruminococcaceae_unclassified,Otu00389 | Reintroduced | -0,000402 | 0,000098 |
| Bacteria,Firmicutes,Clostridia,Clostridiales,Ruminococcaceae,Ruminococcaceae_unclassified,Otu00369 | Reintroduced | -0,000396 | 0,000110 |
| Bacteria,Firmicutes,Clostridia,Clostridiales,Lachnospiraceae,Blautia,Otu00244 | Judicious | -0,000396 | 0,000148 |
| Bacteria,Firmicutes,Clostridia,Clostridiales,Clostridiales_unclassified,Clostridiales_unclassified,Otu00159 | Judicious | -0,000379 | 0,000148 |
| Bacteria,Firmicutes,Clostridia,Clostridiales,Lachnospiraceae,Ruminococcus2,Otu00258 | Judicious | -0,000372 | 0,000107 |
| Bacteria,Firmicutes,Clostridia,Clostridiales,Lachnospiraceae,Blautia,Otu00188 | Judicious | -0,000369 | 0,000121 |
| Bacteria,Bacteria_unclassified,Bacteria_unclassified,Bacteria_unclassified,Bacteria_unclassified,Bacteria_unclassified,Otu00514 | Judicious | -0,000369 | 0,000159 |
| Bacteria,Firmicutes,Clostridia,Clostridia_unclassified,Clostridia_unclassified,Clostridia_unclassified,Otu00340 | Judicious | -0,000368 | 0,000094 |
| Bacteria,Firmicutes,Clostridia,Clostridiales,Ruminococcaceae,Ruminococcaceae_unclassified,Otu00349 | Judicious | -0,000362 | 0,000142 |
| Bacteria,Firmicutes,Clostridia,Clostridiales,Ruminococcaceae,Ruminococcaceae_unclassified,Otu00349 | Reintroduced | -0,000362 | 0,000142 |
| Bacteria,Firmicutes,Clostridia,Clostridiales,Clostridiales_unclassified,Clostridiales_unclassified,Otu00322 | Judicious | -0,000359 | 0,000105 |
| Bacteria,Firmicutes,Clostridia,Clostridiales,Ruminococcaceae,Clostridium_IV,Otu00186 | Reintroduced | -0,000346 | 0,000146 |
| Bacteria,Firmicutes,Clostridia,Clostridiales,Ruminococcaceae,Ruminococcaceae_unclassified,Otu00248 | Judicious | -0,000342 | 0,000147 |
| Bacteria,Firmicutes,Clostridia,Clostridiales,Clostridiales_unclassified,Clostridiales_unclassified,Otu00220 | Judicious | -0,000330 | 0,000122 |
| Bacteria,Firmicutes,Clostridia,Clostridiales,Clostridiales_unclassified,Clostridiales_unclassified,Otu00271 | Judicious | -0,000306 | 0,000094 |
| Bacteria,Firmicutes,Clostridia,Clostridiales,Clostridiales_unclassified,Clostridiales_unclassified,Otu00271 | Reintroduced | -0,000306 | 0,000094 |
| Bacteria,Firmicutes,Clostridia,Clostridiales,Ruminococcaceae,Ruminococcaceae_unclassified,Otu00424 | Judicious | -0,000302 | 0,000072 |
| Bacteria,Actinobacteria,Actinobacteria,Coriobacteriales,Coriobacteriaceae,Coriobacteriaceae_unclassified,Otu00386 | Judicious | -0,000299 | 0,000042 |
| Bacteria,Actinobacteria,Actinobacteria,Coriobacteriales,Coriobacteriaceae,Coriobacteriaceae_unclassified,Otu00386 | Reintroduced | -0,000299 | 0,000042 |
| Bacteria,Firmicutes,Clostridia,Clostridiales,Ruminococcaceae,Ruminococcaceae_unclassified,Otu00353 | Judicious | -0,000292 | 0,000058 |
| Bacteria,Firmicutes,Clostridia,Clostridiales,Ruminococcaceae,Ruminococcaceae_unclassified,Otu00317 | Judicious | -0,000289 | 0,000107 |
| Bacteria,Firmicutes,Clostridia,Clostridiales,Ruminococcaceae,Ruminococcaceae_unclassified,Otu00260 | Judicious | -0,000288 | 0,000100 |
| Bacteria,Firmicutes,Clostridia,Clostridiales,Lachnospiraceae,Lachnospiraceae_unclassified,Otu00432 | Judicious | -0,000273 | 0,000063 |
| Bacteria,Firmicutes,Clostridia,Clostridiales,Ruminococcaceae,Ruminococcaceae_unclassified,Otu00388 | Judicious | -0,000263 | 0,000060 |
| Bacteria,Firmicutes,Clostridia,Clostridiales,Ruminococcaceae,Clostridium_IV,Otu00412 | Judicious | -0,000257 | 0,000091 |
| Bacteria,Firmicutes,Clostridia,Clostridiales,Clostridiales_unclassified,Clostridiales_unclassified,Otu00371 | Reintroduced | -0,000257 | 0,000065 |
| Bacteria,Firmicutes,Clostridia,Clostridiales,Clostridiales_unclassified,Clostridiales_unclassified,Otu00401 | Judicious | -0,000250 | 0,000088 |
| Bacteria,Firmicutes,Clostridia,Clostridiales,Ruminococcaceae,Ruminococcaceae_unclassified,Otu00369 | Judicious | -0,000243 | 0,000110 |
| Bacteria,Firmicutes,Clostridia,Clostridiales,Ruminococcaceae,Ruminococcaceae_unclassified,Otu00422 | Judicious | -0,000234 | 0,000043 |
| Bacteria,Firmicutes,Clostridia,Clostridiales,Ruminococcaceae,Ruminococcaceae_unclassified,Otu00422 | Reintroduced | -0,000234 | 0,000043 |
| Bacteria,Firmicutes,Clostridia,Clostridiales,Ruminococcaceae,Anaerotruncus,Otu00312 | Judicious | -0,000233 | 0,000065 |
| Bacteria,Firmicutes,Clostridia,Clostridiales,Ruminococcaceae,Ruminococcaceae_unclassified,Otu00586 | Judicious | -0,000231 | 0,000051 |
| Bacteria,Firmicutes,Clostridia,Clostridiales,Ruminococcaceae,Ruminococcaceae_unclassified,Otu00408 | Judicious | -0,000230 | 0,000091 |
| Bacteria,Firmicutes,Clostridia,Clostridiales,Ruminococcaceae,Clostridium_IV,Otu00448 | Judicious | -0,000230 | 0,000041 |
| Bacteria,Firmicutes,Clostridia,Clostridiales,Ruminococcaceae,Ruminococcaceae_unclassified,Otu00342 | Judicious | -0,000229 | 0,000112 |
| Bacteria,Firmicutes,Clostridia,Clostridiales,Lachnospiraceae,Lachnospiraceae_unclassified,Otu00393 | Judicious | -0,000227 | 0,000041 |
| Bacteria,Firmicutes,Clostridia,Clostridiales,Lachnospiraceae,Lachnospiraceae_unclassified,Otu00393 | Reintroduced | -0,000227 | 0,000041 |
| Bacteria,Firmicutes,Clostridia,Clostridiales,Ruminococcaceae,Ruminococcaceae_unclassified,Otu00434 | Judicious | -0,000225 | 0,000041 |
| Bacteria,Firmicutes,Clostridia,Clostridiales,Lachnospiraceae,Lachnospiraceae_unclassified,Otu00470 | Judicious | -0,000221 | 0,000034 |
| Bacteria,Firmicutes,Clostridia,Clostridiales,Ruminococcaceae,Oscillibacter,Otu00443 | Judicious | -0,000218 | 0,000083 |
| Bacteria,Firmicutes,Clostridia,Clostridiales,Ruminococcaceae,Ruminococcaceae_unclassified,Otu00361 | Judicious | -0,000212 | 0,000092 |
| Bacteria,Firmicutes,Clostridia,Clostridiales,Ruminococcaceae,Ruminococcus,Otu00363 | Judicious | -0,000187 | 0,000026 |
| Bacteria,Firmicutes,Clostridia,Clostridiales,Ruminococcaceae,Ruminococcus,Otu00363 | Reintroduced | -0,000187 | 0,000026 |
| Bacteria,Firmicutes,Clostridia,Clostridiales,Ruminococcaceae,Ruminococcaceae_unclassified,Otu00360 | Judicious | -0,000184 | 0,000068 |
| Bacteria,Firmicutes,Clostridia,Clostridiales,Lachnospiraceae,Lachnospiraceae_unclassified,Otu00557 | Judicious | -0,000183 | 0,000054 |
| Bacteria,Firmicutes,Clostridia,Clostridiales,Lachnospiraceae,Lachnospiraceae_unclassified,Otu00557 | Reintroduced | -0,000183 | 0,000054 |
| Bacteria,Firmicutes,Clostridia,Clostridiales,Clostridiales_unclassified,Clostridiales_unclassified,Otu00452 | Judicious | -0,000175 | 0,000021 |
| Bacteria,Firmicutes,Clostridia,Clostridiales,Clostridiales_unclassified,Clostridiales_unclassified,Otu00452 | Reintroduced | -0,000175 | 0,000021 |
| Bacteria,Firmicutes,Clostridia,Clostridiales,Ruminococcaceae,Ruminococcaceae_unclassified,Otu00455 | Judicious | -0,000170 | 0,000040 |
| Bacteria,Firmicutes,Clostridia,Clostridiales,Clostridiales_unclassified,Clostridiales_unclassified,Otu00538 | Judicious | -0,000167 | 0,000024 |
| Bacteria,Firmicutes,Clostridia,Clostridiales,Clostridiales_unclassified,Clostridiales_unclassified,Otu00538 | Reintroduced | -0,000167 | 0,000024 |
| Bacteria,Bacteria_unclassified,Bacteria_unclassified,Bacteria_unclassified,Bacteria_unclassified,Bacteria_unclassified,Otu00469 | Judicious | -0,000159 | 0,000069 |
| Bacteria,Bacteria_unclassified,Bacteria_unclassified,Bacteria_unclassified,Bacteria_unclassified,Bacteria_unclassified,Otu00469 | Reintroduced | -0,000159 | 0,000069 |
| Bacteria,Bacteria_unclassified,Bacteria_unclassified,Bacteria_unclassified,Bacteria_unclassified,Bacteria_unclassified,Otu00492 | Judicious | -0,000155 | 0,000031 |
| Bacteria,Bacteria_unclassified,Bacteria_unclassified,Bacteria_unclassified,Bacteria_unclassified,Bacteria_unclassified,Otu00492 | Reintroduced | -0,000155 | 0,000031 |
| Bacteria,Firmicutes,Firmicutes_unclassified,Firmicutes_unclassified,Firmicutes_unclassified,Firmicutes_unclassified,Otu00417 | Reintroduced | -0,000154 | 0,000057 |
| Bacteria,Firmicutes,Clostridia,Clostridiales,Ruminococcaceae,Clostridium_IV,Otu00365 | Judicious | -0,000151 | 0,000041 |
| Bacteria,Firmicutes,Clostridia,Clostridiales,Ruminococcaceae,Ruminococcaceae_unclassified,Otu00445 | Judicious | -0,000149 | 0,000026 |
| Bacteria,Firmicutes,Clostridia,Clostridiales,Ruminococcaceae,Ruminococcaceae_unclassified,Otu00445 | Reintroduced | -0,000149 | 0,000026 |
| Bacteria,Firmicutes,Clostridia,Clostridiales,Ruminococcaceae,Ruminococcaceae_unclassified,Otu00498 | Reintroduced | -0,000147 | 0,000040 |
| Bacteria,Firmicutes,Clostridia,Clostridiales,Clostridiales_unclassified,Clostridiales_unclassified,Otu00486 | Judicious | -0,000147 | 0,000030 |
| Bacteria,Firmicutes,Clostridia,Clostridiales,Clostridiales_unclassified,Clostridiales_unclassified,Otu00419 | Judicious | -0,000146 | 0,000024 |
| Bacteria,Firmicutes,Clostridia,Clostridiales,Ruminococcaceae,Clostridium_III,Otu00435 | Reintroduced | -0,000145 | 0,000035 |
| Bacteria,Firmicutes,Clostridia,Clostridiales,Ruminococcaceae,Ruminococcaceae_unclassified,Otu00496 | Judicious | -0,000145 | 0,000051 |
| Bacteria,Firmicutes,Clostridia,Clostridiales,Ruminococcaceae,Clostridium_III,Otu00462 | Judicious | -0,000141 | 0,000042 |
| Bacteria,Firmicutes,Clostridia,Clostridiales,Ruminococcaceae,Ruminococcaceae_unclassified,Otu00440 | Judicious | -0,000141 | 0,000045 |
| Bacteria,Firmicutes,Clostridia,Clostridiales,Ruminococcaceae,Ruminococcaceae_unclassified,Otu00567 | Judicious | -0,000141 | 0,000024 |
| Bacteria,Firmicutes,Clostridia,Clostridiales,Ruminococcaceae,Ruminococcaceae_unclassified,Otu00567 | Reintroduced | -0,000141 | 0,000024 |
| Bacteria,Firmicutes,Clostridia,Clostridiales,Clostridiales_unclassified,Clostridiales_unclassified,Otu00493 | Reintroduced | -0,000140 | 0,000057 |
| Bacteria,Firmicutes,Clostridia,Clostridiales,Clostridiales_unclassified,Clostridiales_unclassified,Otu00520 | Judicious | -0,000136 | 0,000012 |
| Bacteria,Firmicutes,Clostridia,Clostridiales,Clostridiales_unclassified,Clostridiales_unclassified,Otu00520 | Reintroduced | -0,000136 | 0,000012 |
| Bacteria,Firmicutes,Clostridia,Clostridiales,Ruminococcaceae,Ruminococcaceae_unclassified,Otu00547 | Judicious | -0,000129 | 0,000016 |
| Bacteria,Firmicutes,Clostridia,Clostridiales,Ruminococcaceae,Ruminococcaceae_unclassified,Otu00547 | Reintroduced | -0,000129 | 0,000016 |
| Bacteria,Firmicutes,Clostridia,Clostridiales,Ruminococcaceae,Ruminococcaceae_unclassified,Otu00487 | Judicious | -0,000129 | 0,000031 |
| Bacteria,Firmicutes,Clostridia,Clostridiales,Lachnospiraceae,Clostridium_XlVb,Otu00527 | Judicious | -0,000127 | 0,000020 |
| Bacteria,Firmicutes,Clostridia,Clostridiales,Lachnospiraceae,Clostridium_XlVb,Otu00527 | Reintroduced | -0,000127 | 0,000020 |
| Bacteria,Firmicutes,Clostridia,Clostridiales,Ruminococcaceae,Ruminococcaceae_unclassified,Otu00723 | Judicious | -0,000119 | 0,000025 |
| Bacteria,Firmicutes,Clostridia,Clostridiales,Ruminococcaceae,Ruminococcaceae_unclassified,Otu00723 | Reintroduced | -0,000119 | 0,000025 |
| Bacteria,Firmicutes,Clostridia,Clostridiales,Ruminococcaceae,Ruminococcaceae_unclassified,Otu00592 | Judicious | -0,000115 | 0,000017 |
| Bacteria,Firmicutes,Clostridia,Clostridiales,Clostridiales_unclassified,Clostridiales_unclassified,Otu00495 | Judicious | -0,000112 | 0,000023 |
| Bacteria,Firmicutes,Clostridia,Clostridiales,Clostridiales_unclassified,Clostridiales_unclassified,Otu00495 | Reintroduced | -0,000112 | 0,000023 |
| Bacteria,Firmicutes,Clostridia,Clostridiales,Clostridiales_unclassified,Clostridiales_unclassified,Otu00473 | Judicious | -0,000109 | 0,000023 |
| Bacteria,Firmicutes,Clostridia,Clostridiales,Clostridiales_unclassified,Clostridiales_unclassified,Otu00473 | Reintroduced | -0,000109 | 0,000023 |
| Bacteria,Firmicutes,Clostridia,Clostridiales,Lachnospiraceae,Clostridium_XlVb,Otu00695 | Judicious | -0,000104 | 0,000020 |
| Bacteria,Firmicutes,Clostridia,Clostridiales,Lachnospiraceae,Clostridium_XlVb,Otu00695 | Reintroduced | -0,000104 | 0,000020 |
| Bacteria,Bacteria_unclassified,Bacteria_unclassified,Bacteria_unclassified,Bacteria_unclassified,Bacteria_unclassified,Otu00600 | Judicious | -0,000103 | 0,000016 |
| Bacteria,Bacteria_unclassified,Bacteria_unclassified,Bacteria_unclassified,Bacteria_unclassified,Bacteria_unclassified,Otu00600 | Reintroduced | -0,000103 | 0,000016 |
| Bacteria,Firmicutes,Clostridia,Clostridiales,Ruminococcaceae,Clostridium_IV,Otu00365 | Reintroduced | -0,000100 | 0,000041 |
| Bacteria,Firmicutes,Clostridia,Clostridiales,Ruminococcaceae,Ruminococcaceae_unclassified,Otu00286 | Judicious | -0,000094 | 0,000037 |
| Bacteria,Firmicutes,Clostridia,Clostridiales,Ruminococcaceae,Ruminococcaceae_unclassified,Otu00595 | Judicious | -0,000092 | 0,000019 |
| Bacteria,Bacteria_unclassified,Bacteria_unclassified,Bacteria_unclassified,Bacteria_unclassified,Bacteria_unclassified,Otu00687 | Judicious | -0,000092 | 0,000012 |
| Bacteria,Bacteria_unclassified,Bacteria_unclassified,Bacteria_unclassified,Bacteria_unclassified,Bacteria_unclassified,Otu00687 | Reintroduced | -0,000092 | 0,000012 |
| Bacteria,Firmicutes,Clostridia,Clostridiales,Ruminococcaceae,Ruminococcaceae_unclassified,Otu00521 | Reintroduced | -0,000085 | 0,000018 |
| Bacteria,Firmicutes,Clostridia,Clostridiales,Ruminococcaceae,Ruminococcaceae_unclassified,Otu00521 | Judicious | -0,000085 | 0,000018 |
| Bacteria,Firmicutes,Clostridia,Clostridiales,Ruminococcaceae,Clostridium_IV,Otu00447 | Judicious | -0,000081 | 0,000041 |
| Bacteria,Firmicutes,Clostridia,Clostridiales,Ruminococcaceae,Ruminococcaceae_unclassified,Otu00607 | Judicious | -0,000080 | 0,000009 |
| Bacteria,Firmicutes,Clostridia,Clostridiales,Ruminococcaceae,Ruminococcaceae_unclassified,Otu00607 | Reintroduced | -0,000080 | 0,000009 |
| Bacteria,Firmicutes,Clostridia,Clostridiales,Lachnospiraceae,Lachnospiraceae_unclassified,Otu00470 | Reintroduced | -0,000079 | 0,000034 |
| Bacteria,Firmicutes,Clostridia,Clostridiales,Clostridiales_unclassified,Clostridiales_unclassified,Otu00451 | Judicious | -0,000078 | 0,000035 |
| Bacteria,Firmicutes,Clostridia,Clostridiales,Ruminococcaceae,Ruminococcaceae_unclassified,Otu00548 | Judicious | -0,000073 | 0,000008 |
| Bacteria,Firmicutes,Clostridia,Clostridiales,Ruminococcaceae,Ruminococcaceae_unclassified,Otu00548 | Reintroduced | -0,000073 | 0,000008 |
| Bacteria,Firmicutes,Clostridia,Clostridia_unclassified,Clostridia_unclassified,Clostridia_unclassified,Otu00589 | Judicious | -0,000070 | 0,000007 |
| Bacteria,Firmicutes,Clostridia,Clostridia_unclassified,Clostridia_unclassified,Clostridia_unclassified,Otu00589 | Reintroduced | -0,000070 | 0,000007 |
| Bacteria,Firmicutes,Clostridia,Clostridiales,Ruminococcaceae,Ruminococcaceae_unclassified,Otu00605 | Judicious | -0,000068 | 0,000014 |
| Bacteria,Actinobacteria,Actinobacteria,Coriobacteriales,Coriobacteriaceae,Gordonibacter,Otu00543 | Judicious | 0,000070 | 0,000031 |
| Bacteria,Firmicutes,Clostridia,Clostridiales,Ruminococcaceae,Sporobacter,Otu00579 | Judicious | 0,000071 | 0,000014 |
| Bacteria,Firmicutes,Clostridia,Clostridiales,Clostridiales_unclassified,Clostridiales_unclassified,Otu00576 | Judicious | 0,000073 | 0,000007 |
| Bacteria,Firmicutes,Clostridia,Clostridiales,Ruminococcaceae,Sporobacter,Otu00579 | Reintroduced | 0,000091 | 0,000014 |
| Bacteria,Actinobacteria,Actinobacteria,Coriobacteriales,Coriobacteriaceae,Coriobacteriaceae_unclassified,Otu00466 | Reintroduced | 0,000099 | 0,000026 |
| Bacteria,Firmicutes,Clostridia,Clostridiales,Lachnospiraceae,Lachnospiraceae_unclassified,Otu00598 | Judicious | 0,000108 | 0,000018 |
| Bacteria,Firmicutes,Clostridia,Clostridiales,Lachnospiraceae,Clostridium_XlVb,Otu00578 | Reintroduced | 0,000124 | 0,000023 |
| Bacteria,Firmicutes,Clostridia,Clostridiales,Ruminococcaceae,Ruminococcaceae_unclassified,Otu00438 | Judicious | 0,000132 | 0,000030 |
| Bacteria,Actinobacteria,Actinobacteria,Coriobacteriales,Coriobacteriaceae,Gordonibacter,Otu00476 | Judicious | 0,000134 | 0,000022 |
| Bacteria,Firmicutes,Clostridia,Clostridiales,Clostridiales_unclassified,Clostridiales_unclassified,Otu00518 | Judicious | 0,000154 | 0,000026 |
| Bacteria,Actinobacteria,Actinobacteria,Coriobacteriales,Coriobacteriaceae,Coriobacteriaceae_unclassified,Otu00466 | Judicious | 0,000154 | 0,000026 |
| Bacteria,Bacteria_unclassified,Bacteria_unclassified,Bacteria_unclassified,Bacteria_unclassified,Bacteria_unclassified,Otu00610 | Judicious | 0,000159 | 0,000042 |
| Bacteria,Firmicutes,Firmicutes_unclassified,Firmicutes_unclassified,Firmicutes_unclassified,Firmicutes_unclassified,Otu00343 | Judicious | 0,000174 | 0,000039 |
| Bacteria,Bacteria_unclassified,Bacteria_unclassified,Bacteria_unclassified,Bacteria_unclassified,Bacteria_unclassified,Otu00346 | Reintroduced | 0,000203 | 0,000063 |
| Bacteria,Bacteria_unclassified,Bacteria_unclassified,Bacteria_unclassified,Bacteria_unclassified,Bacteria_unclassified,Otu00488 | Judicious | 0,000212 | 0,000044 |
| Bacteria,Firmicutes,Clostridia,Clostridiales,Ruminococcaceae,Ruminococcaceae_unclassified,Otu00409 | Judicious | 0,000231 | 0,000031 |
| Bacteria,Firmicutes,Clostridia,Clostridiales,Lachnospiraceae,Lachnospiraceae_unclassified,Otu00380 | Judicious | 0,000247 | 0,000102 |
| Bacteria,Firmicutes,Clostridia,Clostridiales,Lachnospiraceae,Lachnospiraceae_unclassified,Otu00501 | Reintroduced | 0,000272 | 0,000101 |
| Bacteria,Firmicutes,Clostridia,Clostridiales,Lachnospiraceae,Lachnospiraceae_unclassified,Otu00378 | Reintroduced | 0,000278 | 0,000052 |
| Bacteria,Firmicutes,Clostridia,Clostridiales,Clostridiales_unclassified,Clostridiales_unclassified,Otu00364 | Judicious | 0,000280 | 0,000081 |
| Bacteria,Firmicutes,Clostridia,Clostridiales,Clostridiales_unclassified,Clostridiales_unclassified,Otu00410 | Judicious | 0,000286 | 0,000055 |
| Bacteria,Firmicutes,Clostridia,Clostridiales,Lachnospiraceae,Lachnospiraceae_unclassified,Otu00284 | Judicious | 0,000312 | 0,000117 |
| Bacteria,Firmicutes,Bacilli,Lactobacillales,Lactobacillaceae,Lactobacillus,Otu00212 | Judicious | 0,000320 | 0,000055 |
| Bacteria,Firmicutes,Clostridia,Clostridiales,Lachnospiraceae,Ruminococcus2,Otu00258 | Reintroduced | 0,000365 | 0,000107 |
| Bacteria,Firmicutes,Clostridia,Clostridiales,Clostridiales_unclassified,Clostridiales_unclassified,Otu00265 | Reintroduced | 0,000375 | 0,000188 |
| Bacteria,Firmicutes,Clostridia,Clostridiales,Ruminococcaceae,Ruminococcaceae_unclassified,Otu00328 | Judicious | 0,000386 | 0,000085 |
| Bacteria,Firmicutes,Clostridia,Clostridiales,Clostridiales_unclassified,Clostridiales_unclassified,Otu00285 | Judicious | 0,000390 | 0,000148 |
| Bacteria,Firmicutes,Clostridia,Clostridiales,Lachnospiraceae,Lachnospiraceae_unclassified,Otu00540 | Judicious | 0,000406 | 0,000084 |
| Bacteria,Bacteria_unclassified,Bacteria_unclassified,Bacteria_unclassified,Bacteria_unclassified,Bacteria_unclassified,Otu00367 | Reintroduced | 0,000427 | 0,000075 |
| Bacteria,Firmicutes,Clostridia,Clostridiales,Clostridiales_unclassified,Clostridiales_unclassified,Otu00285 | Reintroduced | 0,000472 | 0,000148 |
| Bacteria,Firmicutes,Clostridia,Clostridiales,Lachnospiraceae,Lachnospiraceae_unclassified,Otu00243 | Judicious | 0,000473 | 0,000128 |
| Bacteria,Firmicutes,Clostridia,Clostridiales,Lachnospiraceae,Lachnospiraceae_unclassified,Otu00377 | Judicious | 0,000493 | 0,000118 |
| Bacteria,Firmicutes,Firmicutes_unclassified,Firmicutes_unclassified,Firmicutes_unclassified,Firmicutes_unclassified,Otu00203 | Reintroduced | 0,000495 | 0,000116 |
| Bacteria,Bacteria_unclassified,Bacteria_unclassified,Bacteria_unclassified,Bacteria_unclassified,Bacteria_unclassified,Otu00280 | Reintroduced | 0,000539 | 0,000130 |
| Bacteria,Bacteria_unclassified,Bacteria_unclassified,Bacteria_unclassified,Bacteria_unclassified,Bacteria_unclassified,Otu00307 | Reintroduced | 0,000584 | 0,000233 |
| Bacteria,Firmicutes,Clostridia,Clostridiales,Clostridiales_unclassified,Clostridiales_unclassified,Otu00216 | Judicious | 0,000604 | 0,000095 |
| Bacteria,Proteobacteria,Proteobacteria_unclassified,Proteobacteria_unclassified,Proteobacteria_unclassified,Proteobacteria_unclassified,Otu00225 | Reintroduced | 0,000731 | 0,000216 |
| Bacteria,Firmicutes,Clostridia,Clostridiales,Ruminococcaceae,Ruminococcaceae_unclassified,Otu00236 | Reintroduced | 0,000907 | 0,000294 |
| Bacteria,Firmicutes,Clostridia,Clostridiales,Clostridiales_unclassified,Clostridiales_unclassified,Otu00242 | Judicious | 0,001037 | 0,000255 |
| Bacteria,Firmicutes,Clostridia,Clostridiales,Ruminococcaceae,Anaerotruncus,Otu00183 | Reintroduced | 0,001062 | 0,000448 |
| Bacteria,Firmicutes,Clostridia,Clostridiales,Ruminococcaceae,Faecalibacterium,Otu00158 | Reintroduced | 0,001065 | 0,000468 |
| Bacteria,Firmicutes,Clostridia,Clostridiales,Lachnospiraceae,Anaerostipes,Otu00182 | Judicious | 0,001091 | 0,000493 |
| Bacteria,Firmicutes,Clostridia,Clostridiales,Lachnospiraceae,Lachnospiraceae_unclassified,Otu00335 | Judicious | 0,001136 | 0,000464 |
| Bacteria,Firmicutes,Clostridia,Clostridiales,Ruminococcaceae,Ruminococcaceae_unclassified,Otu00163 | Reintroduced | 0,001154 | 0,000213 |
| Bacteria,Firmicutes,Clostridia,Clostridiales,Clostridiales_unclassified,Clostridiales_unclassified,Otu00178 | Judicious | 0,001244 | 0,000248 |
| Bacteria,Firmicutes,Clostridia,Clostridiales,Lachnospiraceae,Clostridium_XlVb,Otu00130 | Judicious | 0,001314 | 0,000218 |
| Bacteria,Firmicutes,Clostridia,Clostridiales,Ruminococcaceae,Ruminococcaceae_unclassified,Otu00180 | Reintroduced | 0,001369 | 0,000488 |
| Bacteria,Firmicutes,Firmicutes_unclassified,Firmicutes_unclassified,Firmicutes_unclassified,Firmicutes_unclassified,Otu00194 | Judicious | 0,001878 | 0,000686 |
| Bacteria,Firmicutes,Clostridia,Clostridiales,Ruminococcaceae,Subdoligranulum,Otu00055 | Judicious | 0,001881 | 0,000570 |
| Bacteria,Firmicutes,Clostridia,Clostridiales,Ruminococcaceae,Faecalibacterium,Otu00158 | Judicious | 0,001932 | 0,000468 |
| Bacteria,Firmicutes,Clostridia,Clostridiales,Ruminococcaceae,Butyricicoccus,Otu00080 | Judicious | 0,002513 | 0,001254 |
| Bacteria,Firmicutes,Clostridia,Clostridiales,Ruminococcaceae,Ruminococcaceae_unclassified,Otu00084 | Judicious | 0,002733 | 0,000743 |
| Bacteria,Firmicutes,Clostridia,Clostridiales,Lachnospiraceae,Lachnospiraceae_unclassified,Otu00174 | Judicious | 0,003322 | 0,001335 |
| Bacteria,Bacteroidetes,Bacteroidia,Bacteroidales,Porphyromonadaceae,Odoribacter,Otu00075 | Reintroduced | 0,003431 | 0,000965 |
| Bacteria,Firmicutes,Clostridia,Clostridiales,Lachnospiraceae,Lachnospiraceae_unclassified,Otu00115 | Judicious | 0,003699 | 0,000854 |
| Bacteria,Firmicutes,Clostridia,Clostridiales,Clostridiales_unclassified,Clostridiales_unclassified,Otu00036 | Judicious | 0,003703 | 0,001057 |
| Bacteria,Firmicutes,Clostridia,Clostridiales,Clostridiales_unclassified,Clostridiales_unclassified,Otu00144 | Judicious | 0,004156 | 0,000822 |
| Bacteria,Firmicutes,Clostridia,Clostridiales,Ruminococcaceae,Ruminococcaceae_unclassified,Otu00079 | Reintroduced | 0,004369 | 0,001594 |
| Bacteria,Firmicutes,Clostridia,Clostridiales,Ruminococcaceae,Butyricicoccus,Otu00044 | Judicious | 0,005212 | 0,000994 |
| Bacteria,Bacteroidetes,Bacteroidia,Bacteroidales,Rikenellaceae,Alistipes,Otu00119 | Reintroduced | 0,005262 | 0,001052 |
| Bacteria,Firmicutes,Clostridia,Clostridiales,Ruminococcaceae,Subdoligranulum,Otu00013 | Judicious | 0,005890 | 0,002883 |
| Bacteria,Firmicutes,Erysipelotrichia,Erysipelotrichales,Erysipelotrichaceae,Clostridium_XVIII,Otu00071 | Judicious | 0,008784 | 0,004251 |
| Bacteria,Bacteroidetes,Bacteroidia,Bacteroidales,Bacteroidaceae,Bacteroides,Otu00009 | Judicious | 0,014459 | 0,007067 |
| Bacteria,Bacteroidetes,Bacteroidia,Bacteroidales,Porphyromonadaceae,Butyricimonas,Otu00039 | Judicious | 0,016226 | 0,001033 |

Table S5. Bacterial members associated with the reintroduction of antibiotics, at sampling time point two, using MaAsLin2 at the genus level. At the end of the 15-month study, drug-free barns from farms C, D, E and F reintroduced the antibiotics. The positively associated genera are significantly more abundant in barns that reintroduced the antibiotics than barns that continued or judiciously used antibiotics. The negatively associated genera are significantly less abundant in barns that reintroduced the antibiotics. Taxonomic assignment at genus level was not possible for unclassified members.

| reintroduction of antibiotics | Taxa | Coefficient | SE |
| --- | --- | --- | --- |
| Positively associated | *Sporobacter* | 0,0001 | 0,0000 |
|  | *Ruminococcus* 2 | 0,0004 | 0,0001 |
|  | *Odoribacter* | 0,0034 | 0,0010 |
| Negatively associated | *Lachnospiraceae* unclassified | -0,0106 | 0,0044 |
|  | *Romboutsia* | -0,0019 | 0,0007 |
|  | *Coriobacteriaceae* unclassified | -0,0003 | 0,0001 |

# Bibliography

1. Charlebois A, Jalbert L-A, Harel J, Masson L, Archambault M. Characterization of genes encoding for acquired bacitracin resistance in Clostridium perfringens. PLoS One. 2012;7(9):e44449.

2. Nagpal R, Ogata K, Tsuji H, Matsuda K, Takahashi T, Nomoto K, et al. Sensitive quantification of Clostridium perfringens in human feces by quantitative real-time PCR targeting alpha-toxin and enterotoxin genes. BMC Microbiol. 2015;15(1):219.

3. Lau CH-F, Li B, Zhang T, Tien Y-C, Scott A, Murray R, et al. Impact of pre-application treatment on municipal sludge composition, soil dynamics of antibiotic resistance genes, and abundance of antibiotic-resistance genes on vegetables at harvest. Sci Total Environ. 2017;587-588:214-22.

4. Knapp CW, Zhang W, Sturm BS, Graham DW. Differential fate of erythromycin and beta-lactam resistance genes from swine lagoon waste under different aquatic conditions. Environ Pollut. 2010;158(5):1506-12.

5. Shibata N, Doi Y, Yamane K, Yagi T, Kurokawa H, Shibayama K, et al. PCR Typing of Genetic Determinants for Metallo-β-Lactamases and Integrases Carried by Gram-Negative Bacteria Isolated in Japan, with Focus on the Class 3 Integron. J Clin Microbiol. 2003;41(12):5407.

6. Bozdogan B, Berrezouga L, Kuo M-S, Yurek DA, Farley KA, Stockman BJ, et al. A new resistance gene, linB, conferring resistance to lincosamides by nucleotidylation in Enterococcus faecium HM1025. Antimicrob Agents Chemother. 1999;43(4):925-9.

7. Tremblay CL, Letellier A, Quessy S, Boulianne M, Daignault D, Archambault M. Multiple-antibiotic resistance of Enterococcus faecalis and Enterococcus faecium from cecal contents in broiler chicken and turkey flocks slaughtered in Canada and plasmid colocalization of tetO and ermB genes. J Food Prot. 2011;74(10):1639-48.

8. Liu Y-Y, Wang Y, Walsh TR, Yi L-X, Zhang R, Spencer J, et al. Emergence of plasmid-mediated colistin resistance mechanism MCR-1 in animals and human beings in China: a microbiological and molecular biological study. Lancet Infect Dis. 2016;16(2):161-8.

9. Maynard C, Fairbrother JM, Bekal S, Sanschagrin F, Levesque RC, Brousseau R, et al. Antimicrobial resistance genes in enterotoxigenic Escherichia coli O149:K91 isolates obtained over a 23-year period from pigs. Antimicrob Agents Chemother. 2003;47(10):3214-21.

10. Soltani M, Beighton D, Philpott-Howard J, Woodford N. Mechanisms of resistance to quinupristin-dalfopristin among isolates of Enterococcus faecium from animals, raw meat, and hospital patients in Western Europe. Antimicrob Agents Chemother. 2000;44(2):433-6.

11. Suzuki MT, Taylor LT, DeLong EF. Quantitative analysis of small-subunit rRNA genes in mixed microbial populations via 5′-nuclease assays. Appl Environ Microbiol. 2000;66(11):4605-14.

12. Gaze WH, Zhang L, Abdouslam NA, Hawkey PM, Calvo-Bado L, Royle J, et al. Impacts of anthropogenic activity on the ecology of class 1 integrons and integron-associated genes in the environment. ISME J. 2011;5(8):1253-61.

13. Marti R, Tien YC, Murray R, Scott A, Sabourin L, Topp E. Safely coupling livestock and crop production systems: how rapidly do antibiotic resistance genes dissipate in soil following a commercial application of swine or dairy manure? Appl Environ Microbiol. 2014;80(10):3258-65.
